# Development of the Inhibitors That Target the PD-1/PD-L1 Interaction—A Brief Look at Progress on Small Molecules, Peptides and Macrocycles

**DOI:** 10.3390/molecules24112071

**Published:** 2019-05-30

**Authors:** Katarzyna Guzik, Marcin Tomala, Damian Muszak, Magdalena Konieczny, Aleksandra Hec, Urszula Błaszkiewicz, Marcin Pustuła, Roberto Butera, Alexander Dömling, Tad A. Holak

**Affiliations:** 1 Faculty of Chemistry, Jagiellonian University, Gronostajowa 2, 30-387 Krakow, Poland; marcintomala87@gmail.com (M.T.); damian.muszak@doctoral.uj.edu.pl (D.M.); madzienkakonieczny@gmail.com (M.K.); pustula.marcin@gmail.com (M.P.); tadholak@uj.edu.pl (T.A.H.); 2 Recepton sp. z o.o, Michala Bobrzynskiego 14, 30-348 Krakow, Poland; ola.hec@op.pl (A.H.); urszula.blaszkiewicz@receptonbiotech.com (U.B.); 3 Department for Drug Design, University of Groningen, A. Deusinglaan 9, AV 9713 Groningen, The Netherlands; r.butera@rug.nl (R.B.); a.s.s.domling@rug.nl (A.D.)

**Keywords:** peptide-based and small synthetic molecule inhibitors, lead optimization, scaffold hopping, PD-1/PD-L1 pathway, rational drug design, cancer immunotherapy, cocrystal structures, structure-activity relationship

## Abstract

Cancer immunotherapy based on antibodies targeting the immune checkpoint PD-1/PD-L1 pathway has seen unprecedented clinical responses and constitutes the new paradigm in cancer therapy. The antibody-based immunotherapies have several limitations such as high production cost of the antibodies or their long half-life. Small-molecule inhibitors of the PD-1/PD-L1 interaction have been highly anticipated as a promising alternative or complementary therapeutic to the monoclonal antibodies (mAbs). Currently, the field of developing anti-PD-1/PD-L1 small-molecule inhibitors is intensively explored. In this paper, we review anti-PD-1/PD-L1 small-molecule and peptide-based inhibitors and discuss recent structural and preclinical/clinical aspects of their development. Discovery of the therapeutics based on small-molecule inhibitors of the PD-1/PD-L1 interaction represents a promising but challenging perspective in cancer treatment.

## 1. Introduction

The programmed cell death protein 1 (PD-1, also known as CD279) belongs to the family of immune checkpoint proteins expressed on the surfaces of various immune cells, including T-cells, B-cells, monocytes, natural killer cells, and dendritic cells [1,2]. The constitutive expression of this protein is associated with the presence of activated tumor-reactive T cells. Anti-PD-1 treatments have demonstrated that therapeutic responses are related to PD-1 being presented on the surface of cytotoxic T-cells in the tumor microenvironment [3].

Structurally, PD-1 is a member of the immunoglobulin (Ig) superfamily; in particular, the immune checkpoint proteins are mostly the members of the B7/CD28 and TNF/TNFR superfamilies [4,5]. The human PD-1 (hPD-1) consists of 288 amino acids and is composed of a single Ig variable-type (IgV) extracellular domain, a transmembrane domain and a cytoplasmic domain [4,5]. The cytoplasmic domain has two tyrosine residues, the tyrosine-based switch motif (ITSM) and the membrane-proximal tyrosine that constitutes an immunoreceptor tyrosine-based inhibitory motif (ITIM) [6,7,8]. The PD-1 protein shares 21–33% sequence identity with CTLA-4 and CD-28, receptors that are also involved in the checkpoint immune system. It exists as a monomeric protein in solution as well as on the cell surface [9]. 

PD-1 binds to one of its two ligands: PD-L1 (also known as B7-H1 and CD274) or PD-L2 (known as B7-DC and CD273). This interaction triggers an inhibitory signal to the activated T cells, which induces T cell apoptosis, anergy, and functional exhaustion (Figure S1, Supplementary Materials) [10]. The expression of PD-L1 is observed in nonlymphoid organs and upregulated in response to activated T cells, antigen-presenting cells (APCs), and nonhematopoietic cells. PD-L1 and PD-L2 share 37% sequence identity and have similar functions and expression profiles [11]. Binding affinities between PD-1/PD-L1 and PD-1/PD-L2 interactions are comparable (K_D_ values: 10.4 and 11.3 nM, respectively), but significant differences in the mechanisms of their interaction with PD-1 were reported [12]. PD-L1 has two IgV- and IgC-like extracellular domains and belongs to the type I transmembrane protein family [4,5]. By binding to PD-1, PD-L1 disrupts TCR signaling and CD-28 co-stimulation and inhibits the anti-CD3-mediated activation of human T cells [13]. The overexpression of PD-L1 and PD-L2 in the tumor microenvironment reduce the body’s immune responses, leading to evasion from immune-cell mediated killing of cancer cells (Figure S1) [14,15,16,17,18]. 

Targeting the immune checkpoint proteins with monoclonal antibodies has become the turning point in cancer treatment (Figure S1, Supplementary Materials) [19,20,21]. The research behind this technique has now been acknowledged with the Nobel Prize in Medicine to James P. Allison and Tasuku Honjo in 2018 [22]. To date, six therapeutic antibodies targeting both PD-1 (nivolumab, pembrolizumab and cemiplimab) and PD-L1 (atezolizumab, avelumab, durvalumab), and the anti-CTLA4 antibody (ipilimumab) have gained the approval of the Food and Drug Administration (FDA) and numerous others are currently undergoing clinical trials [23,24,25]. However, mAbs have several disadvantages such as no oral bioavailability, poor diffusion and permeation profiles correlated with high molecular weights of mAbs. Furthermore, mAbs therapies with their unfavorable pharmacokinetic profiles are related to toxicities and immunogenicity, leading to severe immune-related adverse events (irAEs) with, although rarely, lethal consequences [26].

A promising and highly demanded alternative for the mAbs therapeutics are low-molecular-weight inhibitors, which could serve as inexpensive cancer therapeutics in the future. The field of searching for small molecules targeting the PD-1/PD-L1 interaction has become active since the publication of the structure of the fully human complex of these proteins in 2015 [27]. Several co-crystal structures of antibodies, macrocyclic peptides, and small-molecule PD-1/PD-L1 binders have been published since then [4,5]. Moreover, a significant number of patent applications with small molecules claimed to be active as PD-1/PD-L1 inhibitors have been described [11,28,29,30]. Until now, among all disclosed compounds, only one small molecule inhibitor (CA-170, see below) and a macrocyclic peptide (BMS-986189) have proceeded to clinical trial. Within the last few years, several reviews have summarized the progress on the development of peptidic and small-molecule inhibitors of the PD-1/PD-L1 axis with their structural aspects [4,29,30,31,32,33,34]. Herein, we review the most recent advancements in the field of the design and protein interaction of small-molecule inhibitors of the PD-1/PD-L1 interaction. We describe structure-activity relationship (SAR) aspects of the currently known active compounds and their cocrystal structures. This, we hope, should provide information for designing future drug candidates in cancer immunotherapy.

## 2. Inhibitors of the PD-1/PD-L1 Interaction

### 2.1. Peptides and Peptidomimetics as Inhibitors of the PD-1/PD-L1 Pathway

**Figure 1:**
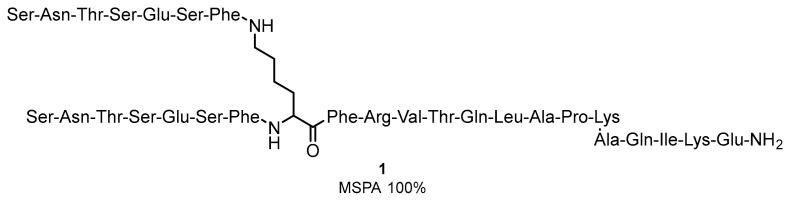
Structure of AUNP-12 (**1**)—A 29-residue peptide sequence.

Peptides and peptidomimetics can be considered the binding bridge between antibodies and small-molecular inhibitors targeting the PD-1/PD-L1 immune checkpoint. The companies Aurigene Discovery Technologies Limited and Pierre Fabre started a cooperation to design new cancer therapeutics in immune-oncology in 2014, which resulted in the structure of AUNP12 (**1**) (Figure 1), an immune checkpoint modulator targeting the PD-1/PD-L1 pathway [33,35]. AUNP-12 is a 29-mer peptide and is highly active in HEK293 cells expressing hPD-L2 (EC_50_ of 0.72 nM). The activity of AUNP-12 was additionally confirmed in the MDA-MB-231-hPD-L1 expressing cells in rat peripheral blood mononuclear cells (PBMC, See Table S1, Figures S2 and S3 in Supplementary Materials for short descriptions of biological assays), leading to the determination of EC_50_ of 0.41 nM in the proliferation assay. AUNP-12 was also reported to inhibit the growth of the B16F10 mouse melanoma cells by 44% and to reduce the 4T1 cells in the mouse breast cancer model. The peptide was also applied to the metastatic lung B16F10 mice cancer cells and in kidney. In contrast to other available therapeutic agents at that time, the compound did not show high toxicity and therefore was the first to be included in clinical trials [36]. The limited SAR studies conducted on the AUNP-12 structure indicated that shortening of the C-terminal chain of these branched peptides and acylation of the side-chain of the N-terminal serine decreases compound activity, whereas acylation of the C-terminal lysine side-chain has no significant impact on the peptide activity.

**Figure 2:**
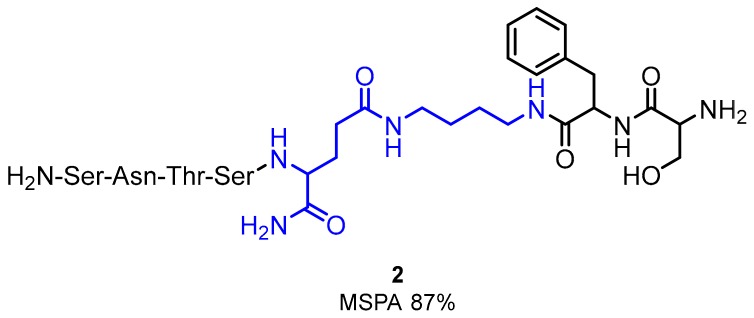
An example of modified heptapeptide (SNTSEFS-NH_2_) derived from **1** (modified moiety is coloured with blue).

Structural modifications of AUNP-12 enabled researchers to select the most active compound **2** (Figure 2)**,** which was tested in mouse splenocyte proliferation assays (MSPA), human peripheral blood mononuclear cell (PBMC) proliferation assays, IFN-γ production in a CLT assay and a response on mice’s cancer cells [28]. The compound **2** was derived from the PD-1 BC loop (Figure 2). In vivo activity of compound **2** in C57BL/6J mice bearing melanoma B16F10 cells exhibited a 64% reduction in lung metastasis at 5 mg/kg [37]. Further SAR studies indicated high tolerance of compound **2** for modifications such as derivatization of the phenyl group with small substituents or *N*-acetylation of the N-terminal amino acids [37].

**Figure 3:**
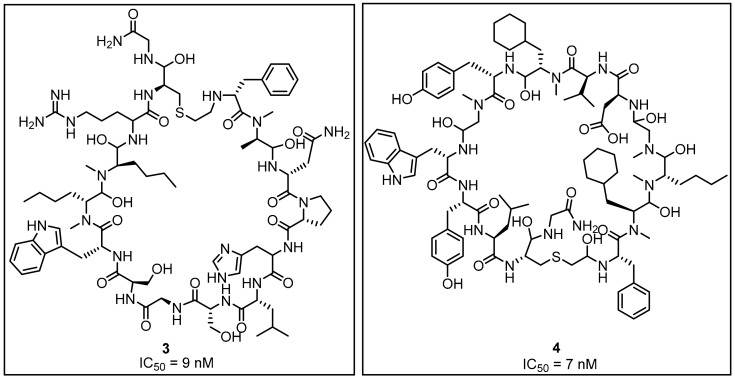
The structures of BMS peptides **3** (BMS-57) and **4** (BMS-71) with their IC_50_ values reported in the patent.

The search for macrocyclic peptides (MCPs) against PD-1/PD-L1 was carried out by Bristol-Myers-Squibb (BMS) [38,39]. An interesting insight into the interaction of macrocyclic peptides and PD-L1 was provided by Magiera-Mularz et al. [40], who described the binding modes and selected aspects of the biological activity of these MCPs. The three peptides (**3**—BMS 57, **4**—BMS 71 and BMS 99—not shown) developed by the BMS company were selected according to the size (15, 14 and 13 amino acid residues) for characterization (Figure 3) [40]. 

**Figure 4:**
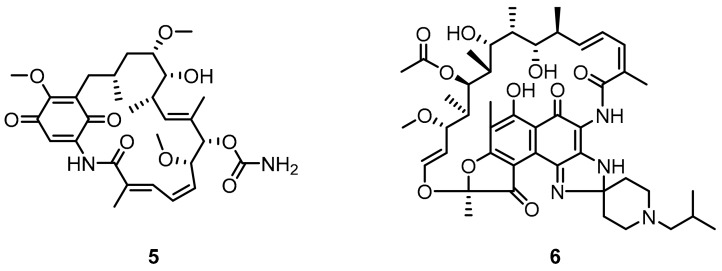
Geldanamycin (**5**) and Rifabutin (**6**) as the PD-L1 inhibitors.

Affinities of BMS-57 and BMS-71 towards PD-L1 were verified with the application of NMR-titration, differential scanning fluorimetry (DSF), and cell-based PD-1/PD-L1 Blockade Bioassay [40]. The NMR titration indicated a lack of activity against PD-1 in these two macrocyclic peptides. A corresponding titration with PD-L1 showed binding affinities of K_d_ values significantly below 0.1 µM. These results were confirmed with the DSF tests and followed by the TCR experiments that showed a dose-dependently restored activity of the TCR responsive promoter. Additionally, the cell-line experiment allowed for determining the EC_50′_s which were 566 nM and 293 nM for BMS-57 and BMS-71, respectively. X-ray structures of the complexes between the peptides and PD-L1 clearly indicated that their pharmacophores differ from those already known for small-molecule compounds [41,42,43]. This may provide an alternative scaffold and extra fragments for the design of new small-molecule antagonists of the PD-1/PD-L1 pathway [38,39,44] (See also Section 2.2, Figure 17). For example, an interesting application of the MCPs as potential inhibitors of PD-L1 was proposed by Patil et al. [45] who showed that macrocyclic compounds **5** and **6** from the ansamycin class of antibiotics are capable to inhibit the PD-1/PD-L1 interaction (Figure 4).

A particularly good result was obtained for **6** that showed an IC_50_ of approximately 25 µM. This led the authors to conclude that such macrocycles could become an inspiration for the development and optimization of a potent and truly small-molecule antagonist of the PD-1/PD-L1 interaction [45]. 

**Figure 5:**
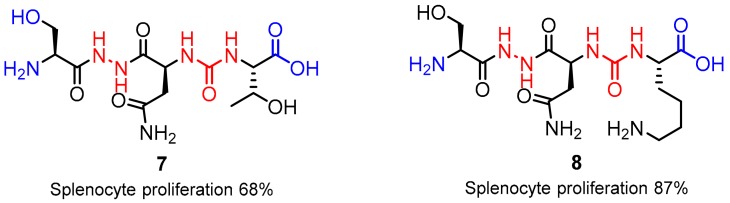
Representative structures of the Aurigene peptidomimetics **7** and **8**. The SAR-affected parts are marked in blue.

A similar approach was carried out by Aurigene, which allowed for designing new peptidomimetics shown in Figure 5. These are tripeptide peptidomimetics with hydrazine and urea linkers as compounds inhibiting the PD-1/PD-L1 interaction. A few structural changes in the proposed scaffold were checked by the Aurigene researchers, which resulted in findings that methylation of both the hydroxy group and the amine group (**7**) at a tip of the structure were not favored [46]; whereas, the coupling of a carboxylic acid moiety with asparagine/glycine residues or its esterification resulted in an increased level of rescue in the MSPA assay (**8**) [28,34,47,48].

Peptidomimetic **7** turned out to be the most active compound showing high activities in the MSPA up to 68% at 100 nM concentration. The compound also showed nanomolar EC_50_ values in the case of both the target proteins PD-L1 (EC_50_ = 30 nM) and PD-L2 (EC_50_ = 40 nM). Subsequent in vivo studies, with application of the CT-26 colon cancer mouse model (3 mg/kg, 25 days) and *Pseudomonas aeruginosa* in a lung infection mice model (10 mg/kg, three times daily, 11 days), showed that **7** is capable of reducing the tumor growth by 46%. The SAR modifications of the scaffold comprised the methylation and amidation of the N-terminal hydroxy and the C-terminal carboxyl correspondingly, which turned out to decrease the compounds activity, whereas the C-terminal threonine carboxyl group methylation was well tolerated (Figure 5). Optimization of the structure mostly focused on the molecules’ amino acid side chains. This led to the discovery of tripeptide peptidomimetics with higher activities, especially when the lysine residue was introduced into the side chain, which led to an increase of the MSPA rates up to 87% [46].

**Figure 6:**
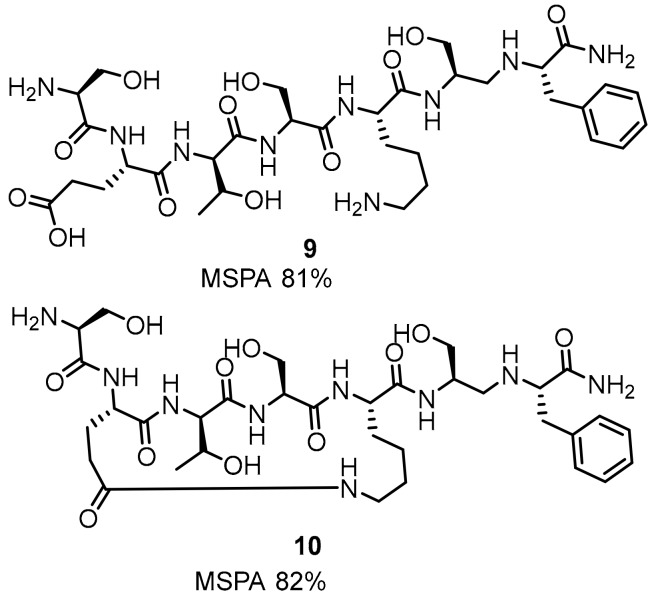
Linear **9** and cyclic peptide **10** showing the highest MSPA rates.

Development of the linear peptides was followed by cyclopeptides and macrocyclic-peptide inhibitors. Closing the peptide with a linker showed increased efficiency of the blockade of the PD-1/PD-L1 interaction. The MSPA assay indicates similar a rescue rate for linear compound **9** and the cyclic compound **10** (Figure 6). 

**Figure 7:**
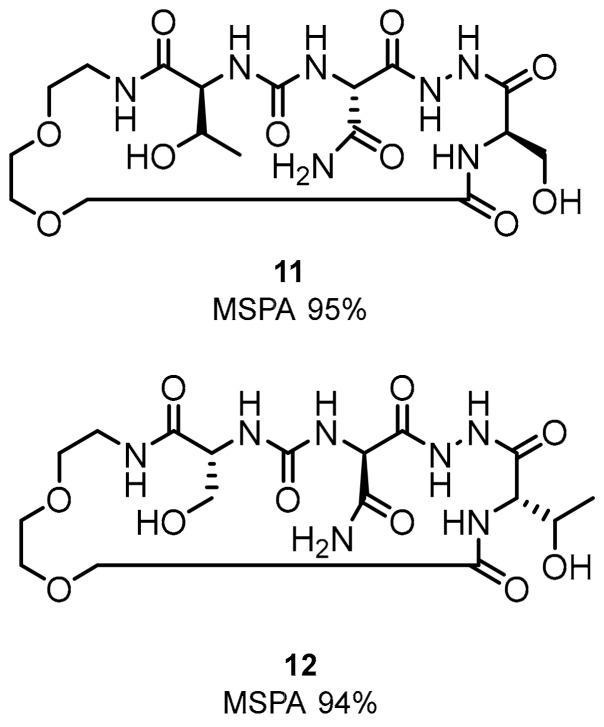
Examples of the macrocyclic peptidomimetic compounds **11** and **12** with glycol-derived linker.

The SAR modifications of the macrocyclic peptides indicated that the compounds with a glycol chain are superior to those with an aliphatic closure [28,33,48]. These modifications were followed by proposing macrocyclic inhibitors **11**, **12** indicating splenocyte proliferation rates of 95 and 94% in the MSPA assay (Figure 7). 

In addition, Aurigene described small-molecules that have activity not only to the PD-1/PD-L1 but also VISTA pathways. The inhibitor CA-170 targeting the PD-1/PD-L1 and VISTA pathway is currently in phase II clinical trial. The compound showed strong activity in animal studies in rodents and mammals, as well as low toxicity [49]. CA-170 also showed successful proliferation and production of IFN-γ. The 2016 clinical trial for advanced oral health treatment has been started, in which CA-170 showed a non-toxic profile. Aurigene together with Curis developed the inhibitor CA-327 for both PD-L1 and T-cell immunoglobulin and mucin domain containing protein-3 (TIM-3). The EC_50_ results were 34 nM (PD-L1) and 35 nM (TIM-3). Additionally, for CA-327, other immune checkpoints (CTLA-4 and VISTA) were not inhibited. This compound shows good modulating properties for the PD-1/PD-L1 pathway comparable to the anti-TIM3 antibody, or the combination of anti-PD-1 antibody and anti-TIM3 antibodies. CA-327 is now under the IND-enabled studies [50]. 

Other tripeptide derivatives proposed by Aurigene are based on the small peptidemimetic compounds (e.g., **7** and **8**, Figure 5) consisting of hydrazine and urea linkers [46]. Significant changes have been made by incorporating 1,2,4-oxadiazole and 1,2,4-thiadiazole, as well as 1,3,4-oxadiazole and 1,3,4-thiadiazole rings in amino acid side chains as the scaffolds for these compounds [51,52].

**Figure 8:**
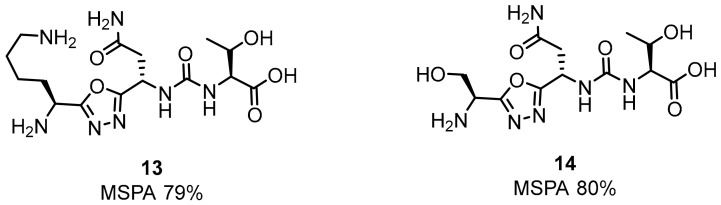
Examples of Aurigene’s small-molecule inhibitors.

Aurigene’s most recent research has been focused on small-molecule inhibitors of structures **13** and **14** shown in Figure 8 [53,54]. 

**Figure 9:**
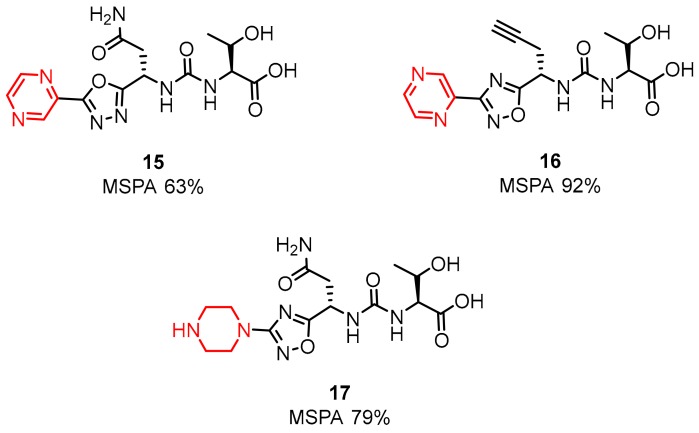
Modifications of structures **13** and **14** [55,56].

The presence of the pyrazine, piperidine and morpholine rings has been shown to contribute to the improvement of the activity of these compounds (Figure 9) [55,56].

**Figure 10:**
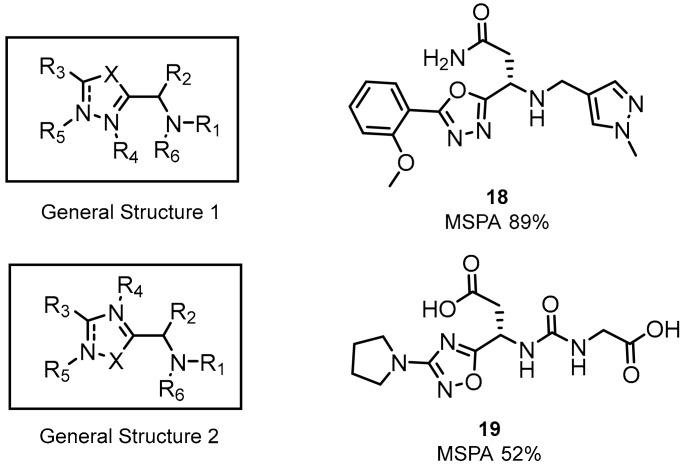
Examples of the small-molecules patented by Aurigene in 2018/2019.

The latest two patents of Aurigene were published at the end of 2018 and early 2019 in which the presented inventions relate to 1,3,4-oxadiazole and 1,3,4-thiadiazole, as well as 1,2,4-oxadiazole and 1,2,4-thiadiazole (Figure 10) [56,57].

**Figure 11:**
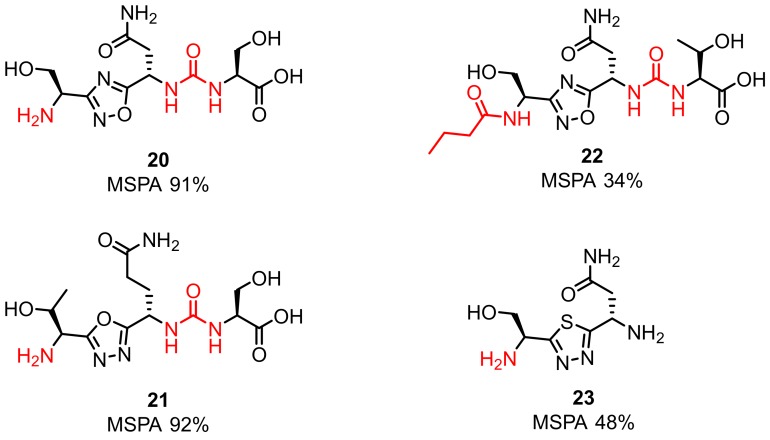
SAR of structures from Aurigene patents [56,57].

These low molecular weight compounds, according to the authors, are therapeutically useful as immune modulators. Greater inhibition activity was observed for compounds with the oxadiazole ring than with the thiadiazole moiety. Additionally, the highest activity was observed for compounds with primary amines and urea moieties in their structure [56,57]. Structures are shown in Figure 11. 

Up to a hundred differently substituted compounds were tested for both the oxa- and thiadiazole scaffolds. The former as well as the latter responded positively for cyclic and aromatic substituents like pyrazine, piperidine or morpholine [34]. The highest splenocyte proliferation in the presence of recombinant mouse PD-L1/PD-L2 (up to 92%) was assigned to the small molecule **21** shown in Figure 11 (**21**) [52,58].

### 2.2. Nonpeptidic Small-Molecule Inhibitors

**Figure 12:**
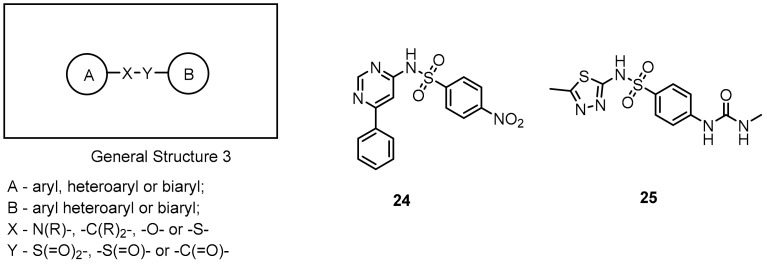
General structure and examples of the PD-1/PD-L1 sulfamonomethoxine **24** and sulfamethizole **25** inhibitors [59].

The first disclosed small-molecule inhibitors targeting the PD-1/PD-L1 axis were derivatives of antibiotics such as sulfamonomethoxines (**24**) and sulfamethizoles (**25**) and were developed by Sharpe et al. (2011). The in vivo efficacy of these compounds was confirmed with the wild type PD-1^C^ T cells and PD-1^−/−^ cells showing their inhibitory activities at the compound concentration in the range of 0–10 µM. The reported compounds influenced rescue of the PD-1-mediated inhibition of INFγ-secretion (in the µM range) in the INFγ-release assay in transgenic mouse T cells expressing PD-1. Moreover, the derivatives **24** and **25** exhibited low cytotoxicity; therefore, they can serve as a template for further antagonist development (Figure 12).

**Figure 13:**
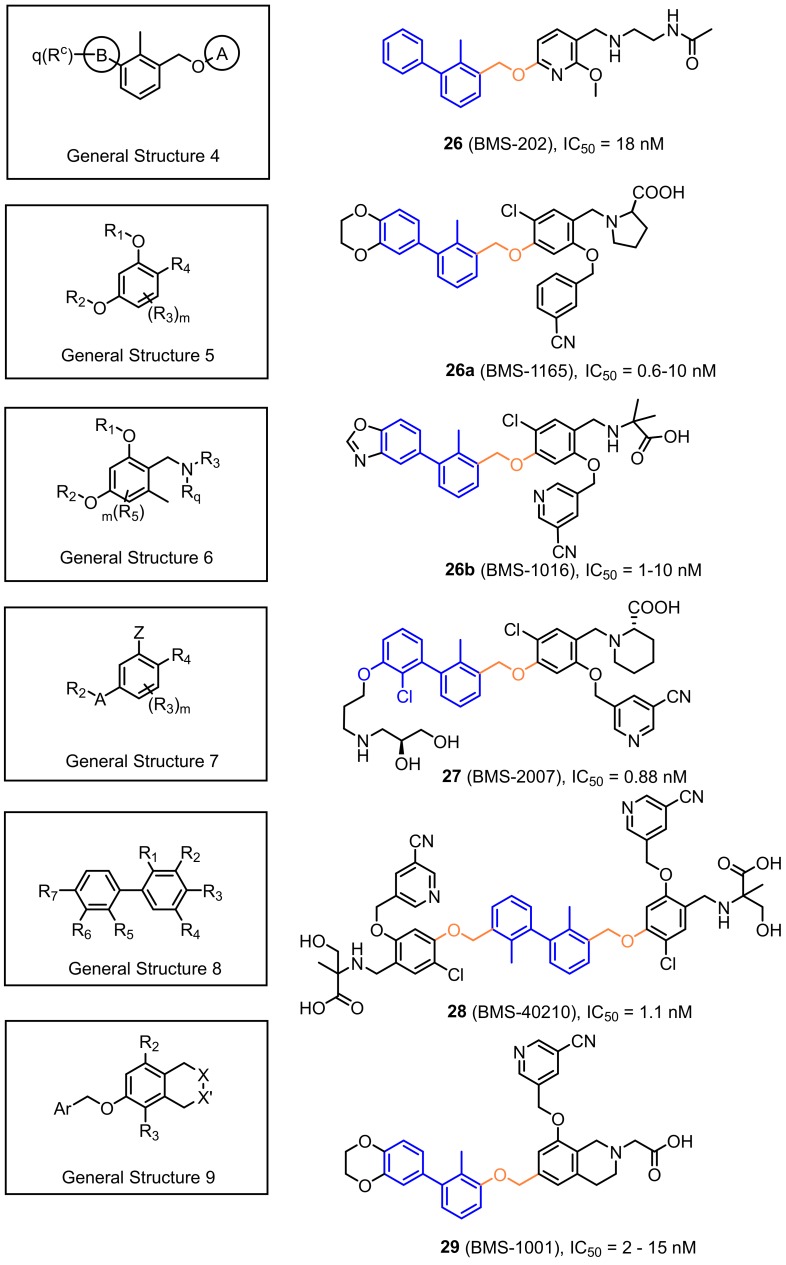
General structures of the compounds disclosed by Bristol-Myers Squibb company and their representative examples [60,61,62,63,64,65].

A significant group of small-molecule inhibitors are the compounds whose scaffold is based on substituted biphenyl group connected to a further aromatic ring through a benzyl ether bond. These series of potent small molecules targeting the PD-1/PD-L1 axis were disclosed in several patents authored by Bristol-Myers Squibb (BMS) (Figure 13, General Structure 4). To determine the binding affinities of these compounds, the authors applied the homogenous time-resolved fluorescence (HTRF) assay with application of the europium cryptate-labeled anti-Ig. The established IC_50′_s are in the range of 0.6 nM up to 20 µM [60,61]. The scope of patent protection was expanded a few months later with the next generation of the compounds with the General Structure 5 [61] (Figure 13). The extended scaffold comprised the replacement of the distal phenyl ring with the 2,3-dihydrobenzo[*b*][1,4]dioxine and addition of cyanopyridine or benzonitrile to the central phenyl, utilizing the benzyl ether bond. These modifications resulted in significant improvement of the binding affinities (values of IC_50_ in the range of 0.6–10 nM according to the HTRF binding assay) [61]. These promising results were further explored and led to the disclosures of even more potent compounds with the General Structures 6–9 (Figure 13). The strategies for improving binding affinities of these compounds concerned the extension of the distal phenyl ring with hydrophilic substituents attached via an ether bond (Figure 13, General Structure 7) or introduction of (pseudo)symmetric biaryl scaffold (Figure 13, General Structure 8) [62,63,64,65]. However, it should be emphasized that none of these patents describes the in vitro or in vivo studies that could provide rationales of the anti-checkpoint protein activity of these compounds. 

**Figure 14:**
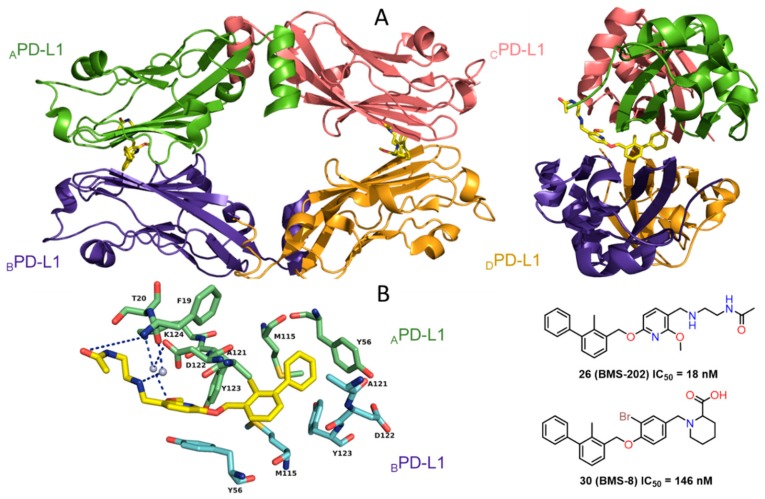
(**A**) Crystal structure of the **26**/PD-L1 complex. The asymmetric unit contains four molecules of PD-L1 (ribbon representation), which are organized into two dimers (_A_PD-L1—green, _B_PD-L1—purple-blue, _c_PD-L1—salmon, _D_PD-L1—bright orange). Each dimer binds a single molecule of **26** (yellow) at the dimer interface. (**B**) Detailed interactions of **26** at the binding cleft of PD-L1 dimer. **26** binds at a hydrophobic cavity formed upon PD-L1 dimerization (PDB: 5J89). Compound **30** cocrystal structure is consistent with structural data for **26**.

The studies by BMS were recently supported by cocrystal structures of compound **26** (BMS-202) and **30** (BMS-8) (PDB: 5J8O and 5J89) [41]. The work provided structural insights into the interactions of the first small-molecules **26**, **30** bound to PD-L1 along with other biochemical data, confirming their inhibitory activities. In contrast to the antibodies, for which the binding to PD-L1 is in a 1:1 stoichiometry, the **26**/PD-L1 ratio in the complex is 1:2 and the asymmetric unit contains four protein molecules, which are organized into two dimers with one inhibitor molecule located at the interface of each dimer (Figure 14).

**Figure 15:**
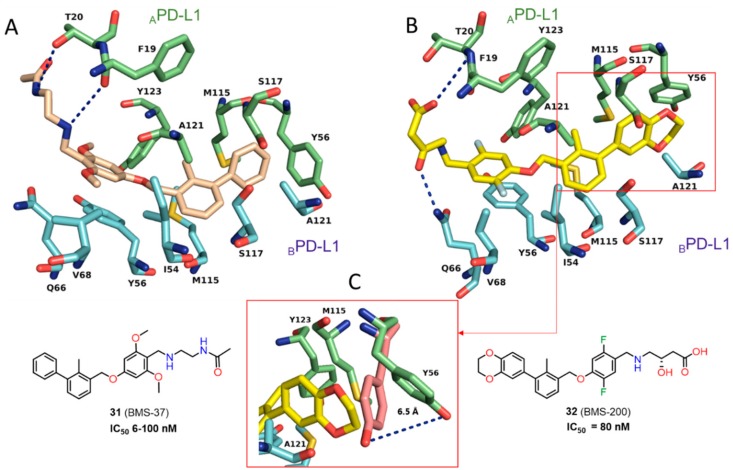
(**A**) Detailed interactions of **31** (light yellow) at the binding cleft of PD-L1. Compound **31** binds at a hydrophobic cavity formed upon PD-L1 binding. (**B**) Detailed interactions of **32** (yellow) at the binding cleft of PD-L1. **32** binds at a hydrophobic tunnel formed upon the PD-L1 dimerization. The movement of the aromatic ring of _A_Tyr56 is induced by the 2,3-dihydro-1,4-benzdioxine moiety. (**C**) The movement of _A_Tyr56 (green) that is induced by the 2,3-dihydro-1,4-benzdioxine group of **32** (yellow) compared with the _A_Tyr56 (salmon) arrangement in the complex of **31**/PD-L1.

Crucial interactions between **26** and PD-L1 involve the formation of T-stacking between the distal phenyl ring from the 2-methylbiphenyl moiety of **26** and the _A_Tyr56 sidechain of PD-L1. This effect is enhanced by the π-alkyl interactions of the methoxy-pyridine group of **26** with the sidechains of _A_Met115 and _A_Ala121 and the π–π stacking with _B_Tyr56. The alkoxypyridine moiety is also responsible for carbonyl-π bonding with _A_Ala121 and finally anion-π bonding with _A_Asp122. The *N*-(2-aminoethyl) acetamide group forms a hydrogen bond to _A_Lys124. The NMR data showed the capability of these compounds to disrupt the PD-1/PD-L1 complex at stoichiometric concentrations (Figure 14) [41]. Two cocrystal structures of **31** (BMS-37) and **32** (BMS-200) (PDB: 5N2D, 5N2F) (Figure 15) were later published revealing new details of the PD-L1 binding interface [42]. Both compounds **31** and **32** are located in the cylindrical, hydrophobic pocket between two hPD-L1 monomers within the dimer interface. Compound **31** shares a similar binding mode to that seen in the previous reports [41], whereas **32** induces the movement of _A_Tyr56 (PD-L1) as the result of its interaction with the 2,3-dihydrobenzo-[b][1,4]dioxane group of the inhibitor (Figure 15). The substituted 2,5-difluorophenyl ring of **31** is stabilized mostly through the π–π stacking interaction with _B_Tyr56s ring with a minor contribution from halogen bonding between a fluorine atom and _A_Asp122. Additionally, (*S*)-4-amino-3-hydroxybutyric acid moiety of **32** creates two hydrogen bonds with _A_Thr20 and _B_Gln66. These conformational changes upon protein binding suggest a flexible character of the PD-L1 binding interface (Figure 15).

**Figure 16:**
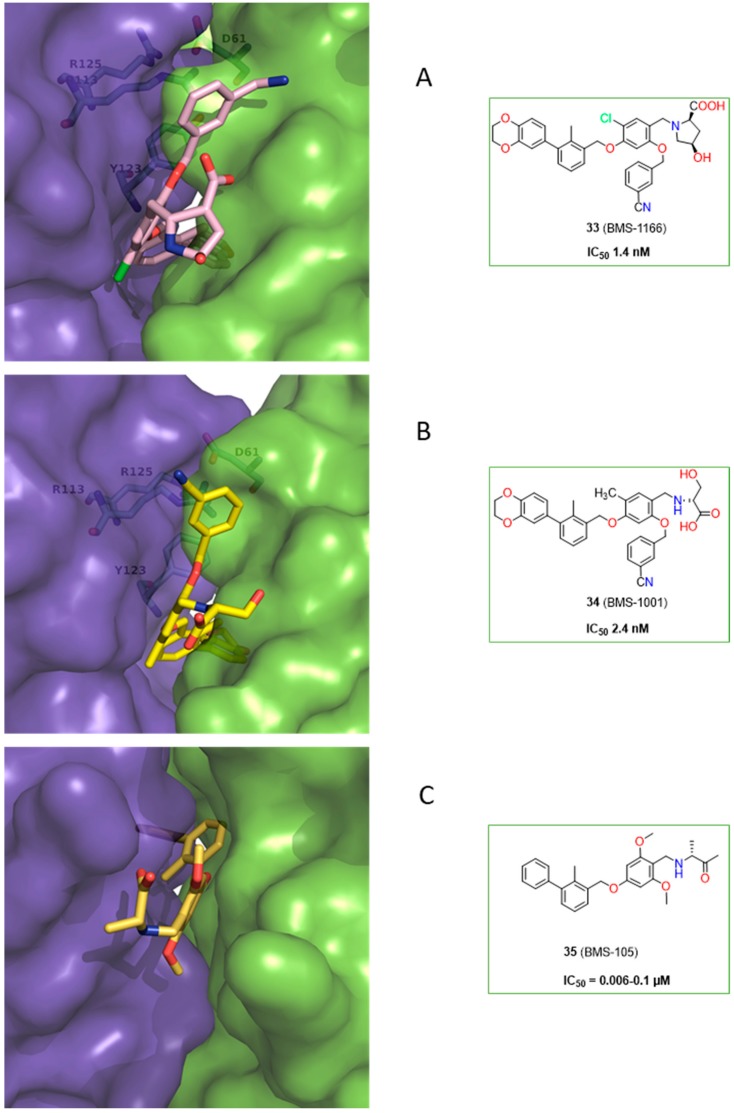
Examples of the BMS class compounds, for which structural data were provided. **(A**, **B**) 2,3-dihydro-1,4-benzodioxine group of **33** and **34** induced transformation of the binding pocket into the binding tunnel across the transverse vertical axis of the dimer. Both compounds trigger formation of a subpocket binding the benzonitrile moiety [41,43,44]. (**C**) The binding cleft of **35** is closed from one side by the _A_Y56 residue. The arrangement of this inhibitor follows patterns for compounds **26**, **30**, and **31**.

Skalniak et al. [43] determined the cocrystal structures of inhibitors **33**, **34** (PDB: 5NIU, 5NIX) along with evaluation of their biological activity. Compounds **33** and **34** exhibited low toxicity to the Jurkat T cells and EC_50_ values of 33.4 and 40.5 µM, respectively. The possibility of restoring the activity of the effector Jurkat T-cells was also indicated for **33** and **34**, however, this effect was lower in comparison to the clinical antibodies. The binding modes for these compounds are comparable with interaction interfaces depicted for **32** and correlated with the position of _A_Tyr56 (PD-L1) determined by the interaction with the 2,3-dihydrobenzo-[b][1,4]dioxine moiety. The main difference is related to the presence of the benzonitrile group, which induces the formation of a subpocket comprised with _B_Arg113, _B_Tyr123, _B_Arg125, and _A_Asp61 (Figure 16). 

**Figure 17:**
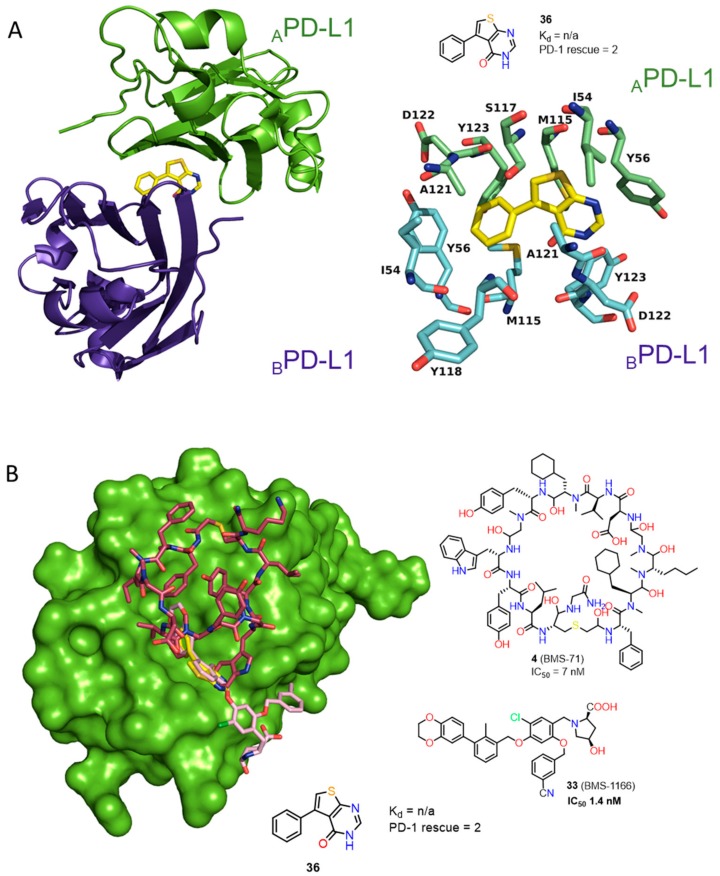
(**A**) Fragment **36** bound to dimeric PD-L1. Detailed interactions of **36** (yellow) at the binding cleft of PD-L1. **36** binds at a hydrophobic tunnel formed upon the PD-L1 dimerization. PDB: 6NM7 (**B**) Overlay of fragment **36** (yellow), BMS small molecule inhibitor **33** (light pink) and BMS macrocyclic inhibitor **4** (plum) (PDB codes: Fragment 1: 6NM7, BMS small molecule 5NIU: 6NM8, BMS peptide-71: 6NNV).

**Figure 18:**
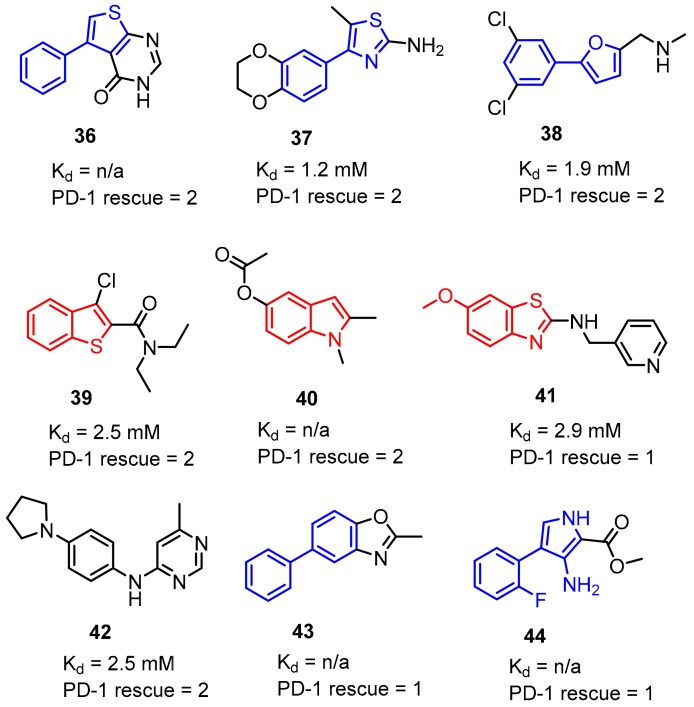
Examples of hits **36**–**44** found in the fragment-based screening published by Perry et al. [44]. Fragments were able to displace PD-1 from PD-L1 in the NMR-based AIDA assay [66]. The PD-1 rescue score was estimated on the percent of the G90 signal rescued of the ^15^N labeled PD-1 at 800 µM fragment concentration. Score 1 indicates 1–15% signal rescue and score 2 indicates >15% signal rescue (some of the K_d_ values were not determined due to solubility limits and/or resonance peak broadening).

More recently Perry et al. [44] reported crystal structures of the macrocyclic peptide **4** (Figure 3, Figure 17) and small molecule inhibitor **35** (Figure 16, Panel B) patented by Bristol-Myers Squibb (BMS) (PDB: 6NNV, 6NM8), which are consistent with the results described recently in the literature [40,41,42,43]. The work of Perry at al. [44] revealed also the results of the NMR fragment-based screening with the PD-L1 IgV domain, leading to identification of 226 hits derived from the library of 13,800 fragments. NMR dissociation assay (AIDA-NMR [66,67,68]) for these hits confirmed the ability of **36** of them to displace PD-1 from the PD-L1/PD-1 complex. These structural fragments (See examples **36**–**44**, Figure 18) can be useful building blocks for the further development of PD-L1 inhibitors.

Fourteen co-crystal structures of fragments bound to the dimeric PD-L1 were determined by Perry et al., which were similar to those found in previously reported structural data for PD-L1 inhibitors (Figure 17A,B) [41,42,43,44]. 

Several types of the biphenyl-based small-molecule inhibitors of the PD-1/PD-L1 interaction were proposed by Incyte Corporation (**45**–**63**). To validate the binding affinities of these compounds, the authors utilized the homogenous time-resolved fluorescence (HTRF) assay with the recombinant PD-L1 protein. 

**Figure 19:**
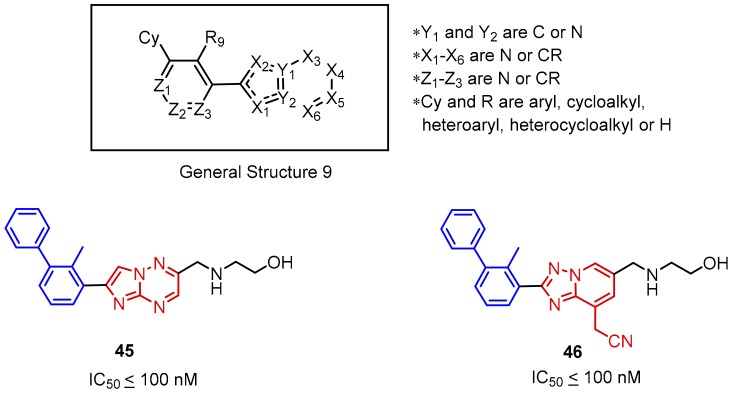
The general structure and examples of the PD-1/PD-L1 inhibitors (**45** and **46**) reported by Incyte Corporation. The biphenyl fragment is shown in blue and the fused heteroaromatic system is shown in red. The next figure follows the same color pattern unless stated otherwise.

The first Incyte scaffold contains a fused heteroaromatic ring attached to the adjacent part of the biphenyl core (Figure 19, General Structure 9). Expansion of this scaffold led to the molecules that contain one to four nitrogen atoms in the fused rings system. The SAR studies showed that an increasing number of nitrogen atoms present in the conjugated ring system and the type of the X_4_ substituent have the strongest influence on the potency of these compounds. The compounds showed nanomolar affinities (the IC_50_ values are in the range of IC_50_ ≤ 100 up to 10,000 nM as determined in the HTRF assay). The most promising data were reported for compounds **45**–**46** with the attached 2-benzylaminoethan-1-oyl moiety in the position X_4_ (IC_50_ < 100 nM) (Figure 19) [69].

**Figure 20:**
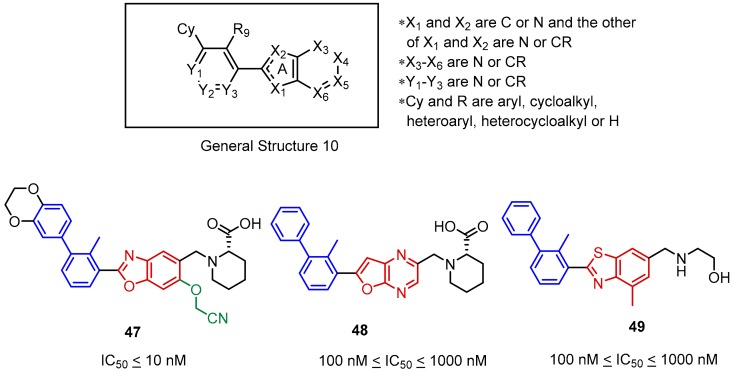
General structure and examples of PD-1/PD-L1 inhibitors (**47**–**49**) reported by Incyte Corporation in the second patent. Aryl ether fragment is shown in green.

The second Incyte disclosure further explored Scaffold 9 (Figure 20, General Structure 10) focusing on the SAR related to the type of heteroatoms that are present in the fused heterocyclic ring system [70]. Therefore, the compounds based on the fused ring systems such as benzoxazole (**47**), furo [2,3-*b*]pyridine (not shown), furo [2,3-*b*]pyrazine (**48**) and benzothiazole (**49**) were tested leading to the conclusion that replacement of carbon atoms with nitrogen at the X_3_ or X_6_ positions, as well as introducing sulphur in the X_2_ position are less favored by SAR. Further studies showed that linking an additional substituent via an ether bond at the X_5_ position (**47**) improves the potency of the compounds. An analogical SAR strategy to that depicted in Figure 20 is very common in the BMS compounds and their modifications (See Figure 13).

**Figure 21:**
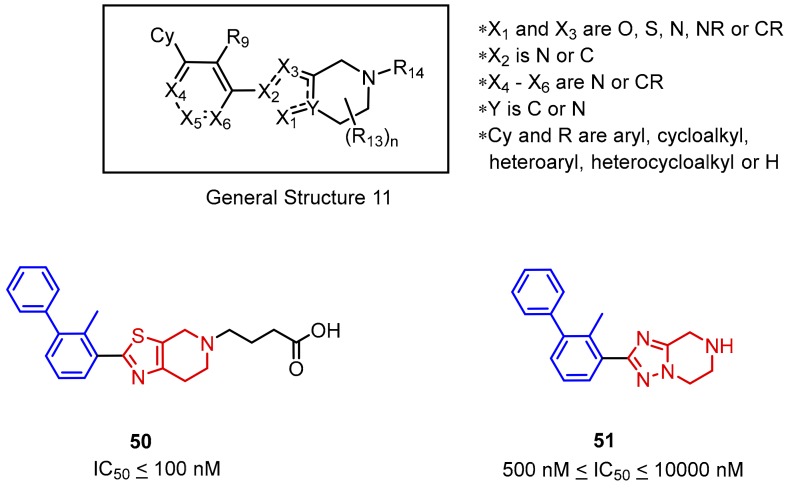
General structure and examples of the PD-1/PD-L1 inhibitors (**50** and **51**) reported by Incyte Corporation [71].

Incyte Corporation also reported scaffold 11 (Figure 21, General Structure 11) comprised of a five-membered heteroaromatic ring fused to a piperidine and connected with the adjacent part of a biphenyl moiety (Figure 21). According to the HTRF assay, indicated IC_50′_s values range from the nanomolar to micromolar range. The SAR tolerates thiazoles, oxazoles, and imidazoles in the role of the fused five-membered ring (Figure 21, compound **50**). A particular decrease in the activity (IC_50_ in the micromolar range) can be observed in the case of X_1_ and Y replacement with a nitrogen atom (Figure 21, **51**) [71].

**Figure 22:**
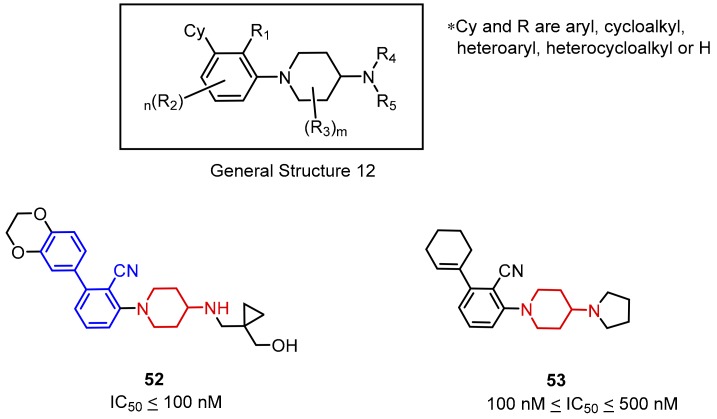
General structure and examples of the PD-1/PD-L1 inhibitors (**50** and **51**) reported by Incyte Corporation [71].

The next Incyte scaffold is based on an *N*-arylated-4-amino piperidine moiety. According to the HTRF results, activities of these compound expressed as IC_50′_s were between 100–500 nM (Figure 21). *N*-arylated-4-aminopiperidine derivatives without the biphenyl moiety (**52**) were also reported. The substitution of the aromatic ring in the biphenyl system with cyclohexene **53** or cyclohexane moieties results in a significant decrease of the compounds’ potency (Figure 22) [72].

**Figure 23:**
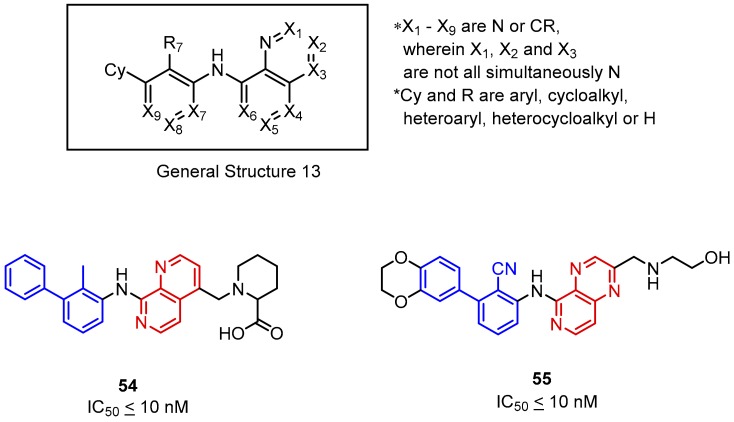
General structure and examples of the PD-1/PD-L1 inhibitors (**54** and **55**) reported by Incyte Corporation, based on the fused six-membered heteroaromatic rings linked with the biphenyl scaffold.

Further research made on small molecules targeting the PD-1/PD-L1 axis led Incyte Corporation to develop a novel group of inhibitors (Figure 23, General Structure 13). The compounds **54** and **55** were based on the biphenyl scaffold linked via an amine bond to an annellated heteroaromatic ring system containing up to three nitrogen atoms. The IC_50_ values of the most active compounds were reported below 10 nM [73]. The potency of these compounds is negatively affected by the absence of nitrogen in the X_6_ position. The reduction of activity was reported also for compounds with a nitrogen atom at the X_4_ position (Figure 23).

**Figure 24:**
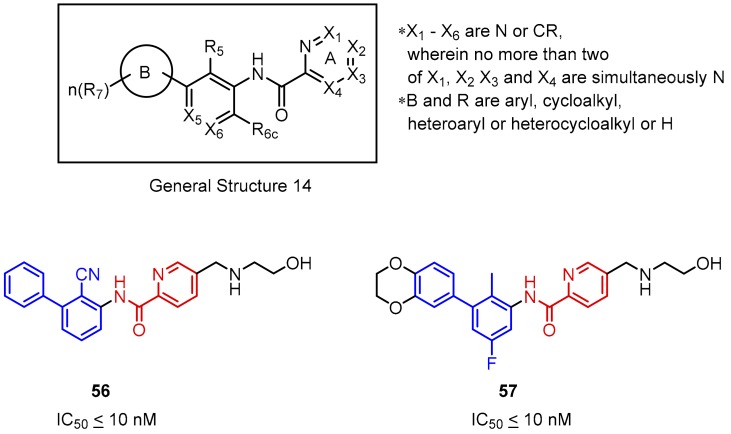
General structure and examples of the PD-1/PD-L1 inhibitors (**56** and **57**) reported by Incyte Corporation, based on the picolinamide-biphenyl scaffold.

Another patent, released by Incyte Corporation comprises molecules derived from picolinamides **56** and **57** linked to the proximal phenyl ring of the biphenyl group (Figure 24). The molecule structure is similar to the previously described scaffold (examples **54** and **55, Figure 23**) containing the six-membered heteroaromatic system. The main difference is in the possibility of rotation in the pyridine-based system during the interaction with the target protein, which may affect the binding strength. However, the IC_50_ values of compound **54** and **55** are comparable to **56** and **57** [74]. It was observed that both, introducing the second nitrogen atom to the pyridine moiety and the presence of a bulky substituent at the X_2_ position (2-benzylaminoethan-1-oyl moiety is favoured) negatively affect the affinities of the compounds with the General Structure 14 (Figure 24).

**Figure 25:**
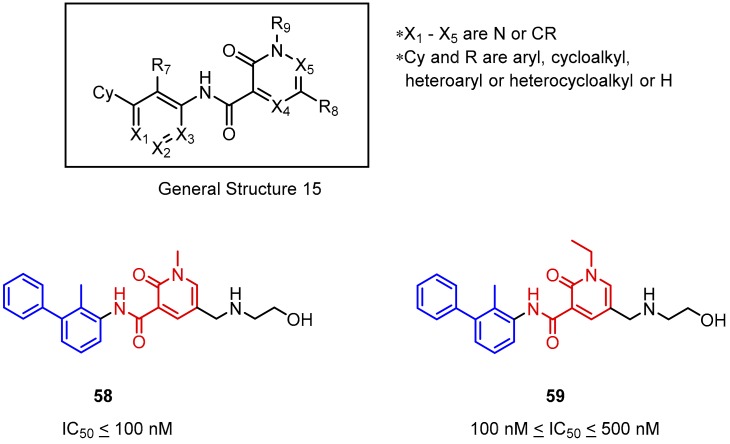
General structure and examples of the PD-1/PD-L1 inhibitors (**58** and **59**) reported by Incyte Corporation and based on the *N*-methyl-2-pyridone-6-carboxamide moiety (depicted with red).

As a continuation of optimization by Incyte Corporation the *N*-methyl-2-pyridone-6-carboxamide derivatives were investigated (General Structure 15, Figure 25). The effect of an oxygen atom in the heteroaromatic system was examined [75]. The results indicate that introduction of a carbonyl function vicinal to the nitrogen atom in pyridine derivatives **58** and **59** has a negative influence on the compound potency in comparison to compounds **56** and **56** (IC_50_ values are about 10 times lower). Moreover, changing the *N*-alkyl substituent from a methyl to an ethyl group results in further reduction of the activity of **58** and **59** (Figure 25).

**Figure 26:**
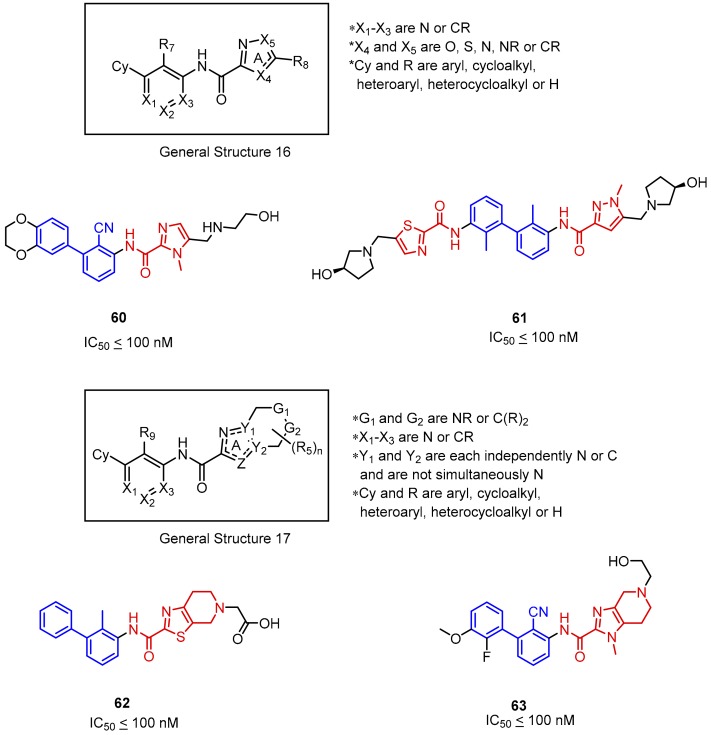
General structures and examples of the PD-1/PD-L1 inhibitors patented by Incyte Corporation, based on heterocyclic five-membered aromatic rings (**60** and **61**) and on heterocyclic five-membered aromatic rings fused with piperidine (**62** and **63**).

Yet another set of molecules authored by Incyte Corporation consists of compounds based on biphenyl and diheterocyclic five-membered aromatic rings joined by an amide bond (General Structure 16, Figure 26). The IC_50_ values were determined with the HTRF assay and were approximately below 100 nM for the best molecules in both series. Interestingly, the elongation at the 3′ position in the biphenyl (Figure 26, **61**) did not cause a decrease in activity as might be inferred after having considered the crystal structure of the PD-L1/**32** complex (See Figure 15) [42]. The compounds based on pyrrole, imidazole, pyrazole, thiazole and oxazole moieties were favored by the SAR; merely pyrroles and oxazoles incorporated into the scaffold were less potent [76,77].

**Figure 27:**
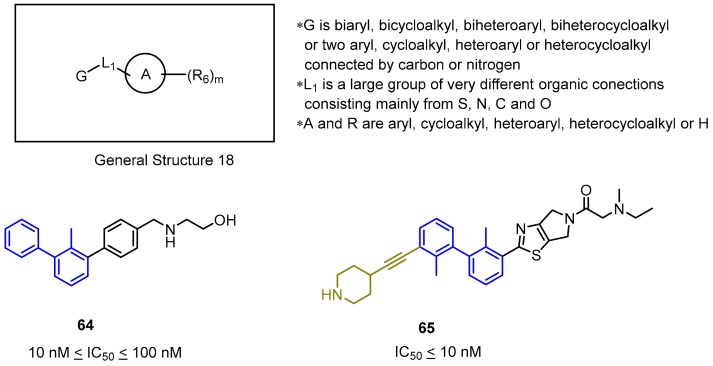
General structure and examples of the PD-1/PD-L1 inhibitors patented by Incyte Corporation (**64** and **65**). The 3′ biphenyl’s substituent is depicted in gold.

The last Incyte patent seems to be a combination of the previously published ones and covers a wide group of rigidified scaffolds based on the biphenyl core fused with a phenyl (**64**) or a heterocyclic ring system (**65**) (General Structure 18, Figure 27), connected together directly (**64**–**65**) or through an amine/amide bond (not shown). The published results show clearly that elongation of the distal phenyl ring in the biphenyl moiety (**65**) has a positive impact on the SAR. The IC_50_ values for the most potent compounds are below 10 nM, which was determined by the HTRF assay [78].

**Figure 28:**
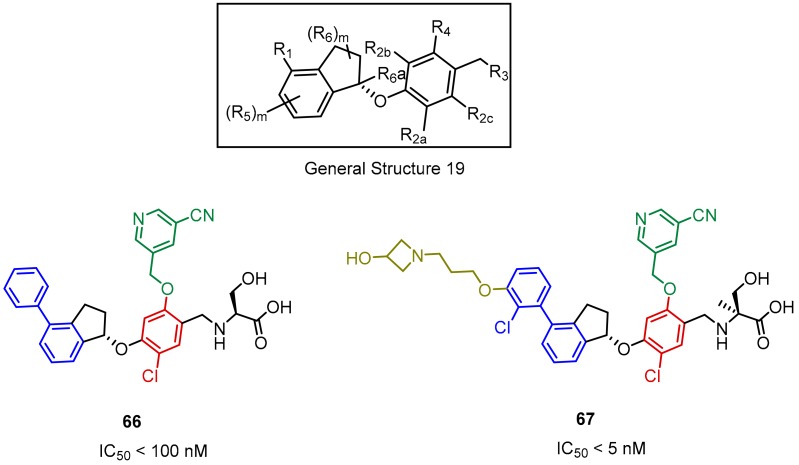
The structures of inhibitors **66**, **67** disclosed by ChemoCentryx.

ChemoCentryx has identified 4-phenyl-2,3-dihydro-1*H-*inden-1-ol derivatives as inhibitors of the PD-1/PD-L1 interaction through rational drug design considerations (General Structure 19, Figure 28). The scaffold follows a similar design pattern as the compounds of Incyte (Figure 19, Figure 20, Figure 21, Figure 26 and Figure 27) with a characteristic rigidified linker, which is composed of a substituted indanyl group fused to the biphenyl moiety. A novel enzyme-linked immunosorbent ELISA assay, measuring inhibition of the PD-1/PD-L1 interaction, followed by a functional cell-based reporter and mixed lymphocyte reaction (MLR) assays were used to detect the activity of these compounds. Moreover, to test in vivo efficacy of the inhibitors, the A375 human melanoma cells along with human peripheral blood mononuclear cells (PBMCs) were co-implanted into the immunodeficient NOD/SCID mice. The identified lead structure CCX4503 inhibited tumor growth in vivo with efficacy comparable to the positive control (anti-human PD-L1 antibody). The disclosed PD-L1 antagonists exhibited IC_50_ values in the nanomolar ranges, but compounds with attached cyanopyridine and substituent at 3′ position of the biphenyl ring were more potent (**67**, Figure 28). The *S*-enantiomers of the inhibitors were more favored by the SAR [79,80,81].

**Figure 29:**
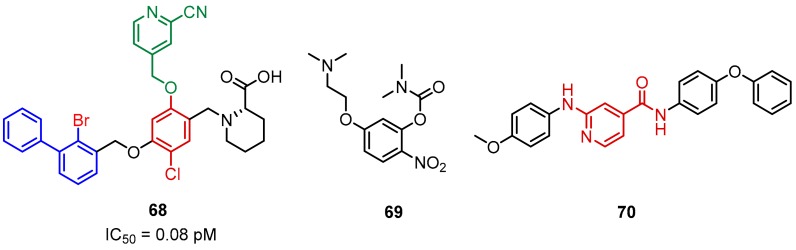
Examples of the benzyl phenyl ether derivatives **68** reported by Feng et al., [82]; the resorcinol (**69**) and isonicotinic acid (**70**) derivatives reported, respectively, by Li et al. [83] and Sun et al. [84].

A series of potent benzyl phenyl ether derivatives (Figure 29, **68**) was reported by Feng and co-workers from the Institute of Materia Medica of Beijing (Chinese Academy of Medical Sciences) [82]. The series of these PD-1/PD-L1 inhibitors have IC_50_ values in the low nanomolar range, according to the HTRF assay conducted with the Cisbio PD-1/PD-L1 binding assay kit. This activity improvement can be rationalized by the introduction of the substituted pyridine methylene moiety and the bromine substituent into the scaffold structure [82]. The representative compound **68** showed an IC_50_ value of 0.08 pM (Figure 29).

The inhibitors based on the resorcinol scaffold have been developed by Li et al. [83] from the Beijing Institute of Pharmacology and Toxicology. The example compound **69** inhibited the PD-1/PD-L1 interaction in 43% at 500 μM in the HTRF binding assay (Figure 29, **69**).

Sun’s group from China Pharmaceutical University had identified the isonicotinic acid derived inhibitors (Figure 29, **70**). The activities of these compounds, discovered by computer-aided drug design (CADD), were established using the HTRF assay. The representative compound **70** induced 36% inhibition of PD-1/PD-L1 binding at a concentration of 10 μM (Figure 29) (Sun et al., 2017).

**Figure 30:**
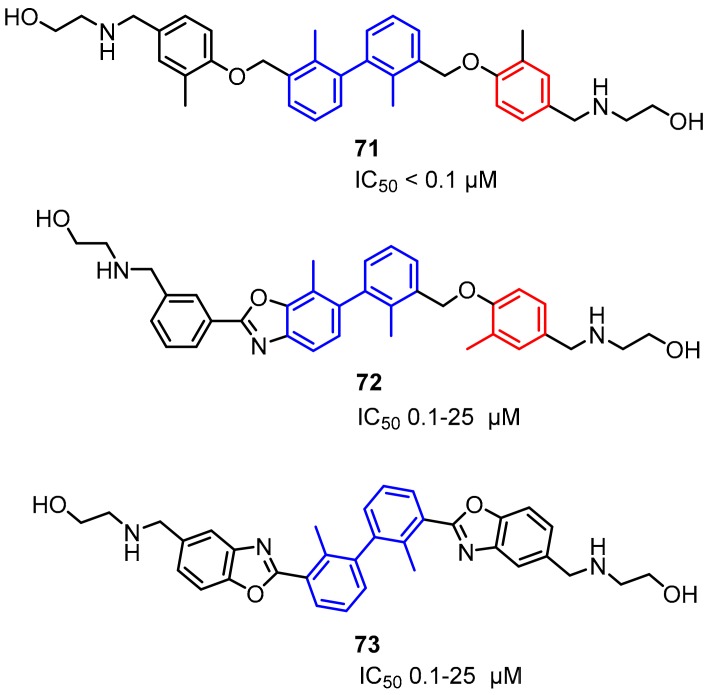
Examples of the structures patented by Arising International LLC.

Arising International LLC reported (pseudo)symmetric compounds **71**–**73** based on the biaryl core as inhibitors of PD-1/PD-L1 and CD80/PD-L1 PPIs [85]. According to the docking experiments, the scaffold binds to a dimeric form of PD-L1, and therefore is capable of dissociating both the PD-1/PD-L1 and CD-80/PD-L1 complexes. The binding affinity of these compounds was assessed on the extracellular domains of PD-1 and PD-L1 utilizing the HTRF binding assay (IC_50_ values from 0.1 to 25 µM) (Figure 30).

**Figure 31:**
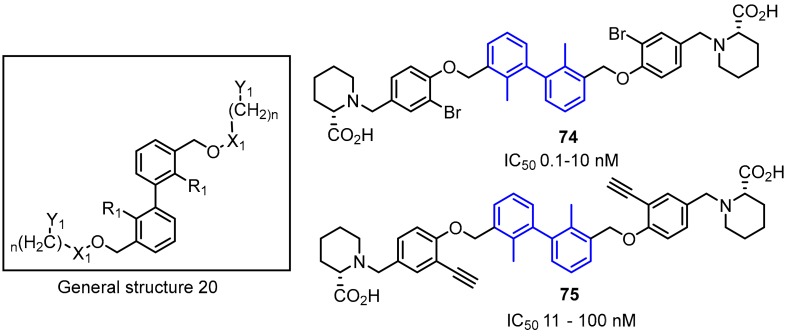
Examples of inhibitors **74**–**75** designed by Polaris Pharmaceuticals company [86].

Polaris Pharmaceutical Inc. revealed a patent which disclosed small-molecules directed to the PD-1/PD-L1 axis [86]. The patented compounds are pseudo-symmetrical, biphenyl derivatives, containing a system of four aromatic rings (General Structure 20, Figure 31). The biological activity of these antagonists was determined with the ELISA (Enzyme-Linked Immunosorbent Assay) and 12 of these compounds exhibited an IC_50_ value in the range of 0.1–10 nM. The substitution of the bromine attached to the phenyl moiety (**74**) with an acetylene group (**75**) resulted in a 10-fold decrease in binding affinities to the target PD-L1 protein [58].

**Figure 32:**
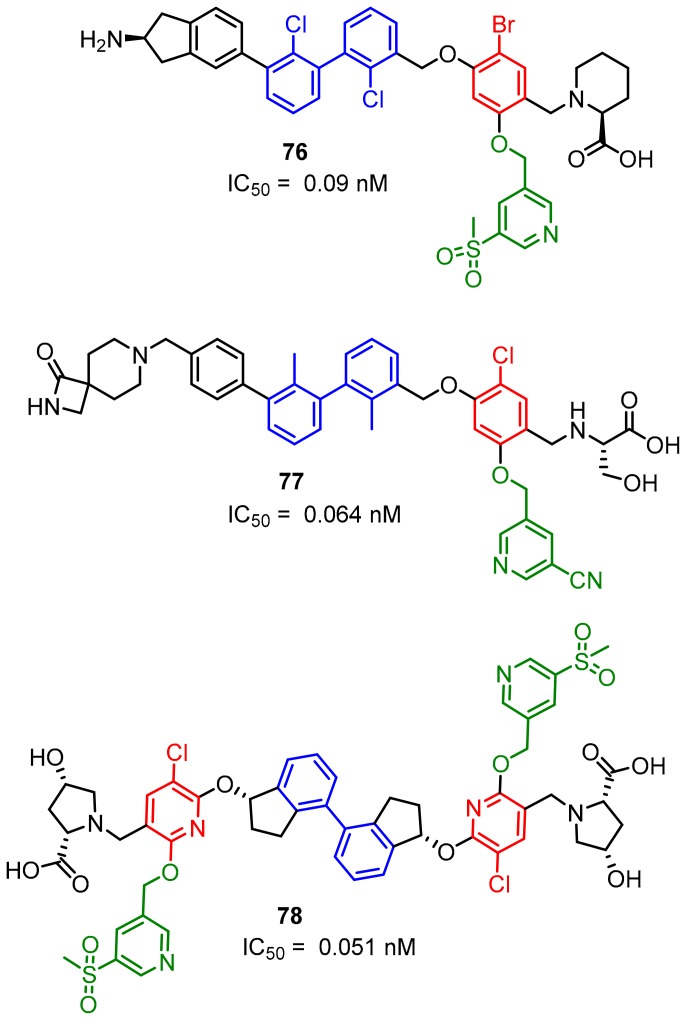
Compounds reported by Aktoudianakis et al. [87] (Gilead Sciences).

Gilead Sciences Inc. designed a series of potent compounds (examples **76**–**78**) with IC_50_ values in the nanomolar range. IC_50′_s were established with the Amplified Luminescent Proximity Homogeneous Assay (ALPHA) (Figure 32) [87]. The majority of compounds inhibited or specifically blocked the interaction between the recombinant extracellular domain of PD-1 and the native cell-bound PD-L1. Moreover, the patent also describes a biochemical assay determining if these inhibitors interact with PD-L1 by dimerizing the extracellular domain of this protein. The disclosed scaffold shares the biaryl core in the central part of the molecule with the series of inhibitors previously described by Polaris Pharmaceuticals (Figure 31) [86], Bristol-Myers-Squibb (Figure 13), Arising International (Figure 30) [85] and Incyte Corporation (Figure 26, compound **61**) [76,77].

**Figure 33:**
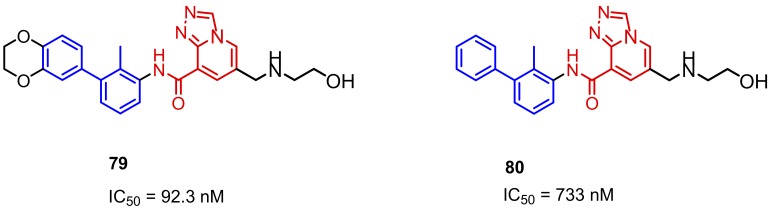
Examples of the compounds published by Qin et al. [88].

More recently, Qin and colleagues published a scaffold based on [1,2,4]triazolo[4,3-*a*]pyridines as the inhibitors of the PD-1/PD-L1 interaction (examples **79** and **80**, Figure 33). The compounds were designed with docking analysis comprising a ring fusion strategy and structure–activity relationship (SAR) studies. These two approaches led to the identification of the promising lead compound **79** with an IC_50_ value of 92.3 nM established with the HTRF binding assay. Additionally, **79** induced the IFN-γ secretion in a T cell-tumor co-culture model of the Hep3B/OS-8/hPD-L1 and CD3 T cells [88].

**Figure 34:**
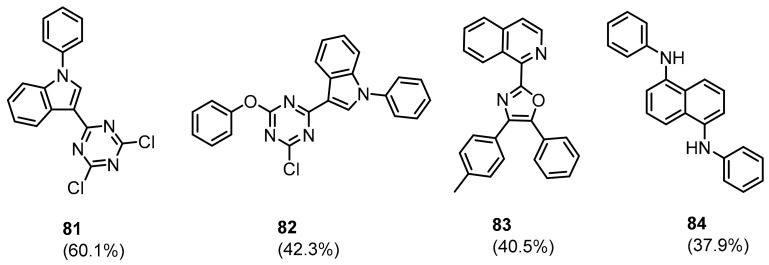
Structures of PD-1 binders reported by Patil and co-workers [89].

Patil and colleagues [89] proposed PD-1/PD-L1 pathway binders using a virtual screening approach based on the X-ray crystal structure of the human PD-1/PD-L1 complex [27]. The 40 most promising hits, found by virtual screening of the National Cancer Institute Diversity database [90], were evaluated with the AlphaLISA™ human PD-1/PD-L1 binding assay. The assay distinguished four weak PD-1 binders **81**–**84** showing ≥30% activity at the concentration of 50 mM (Figure 34).

**Figure 35:**
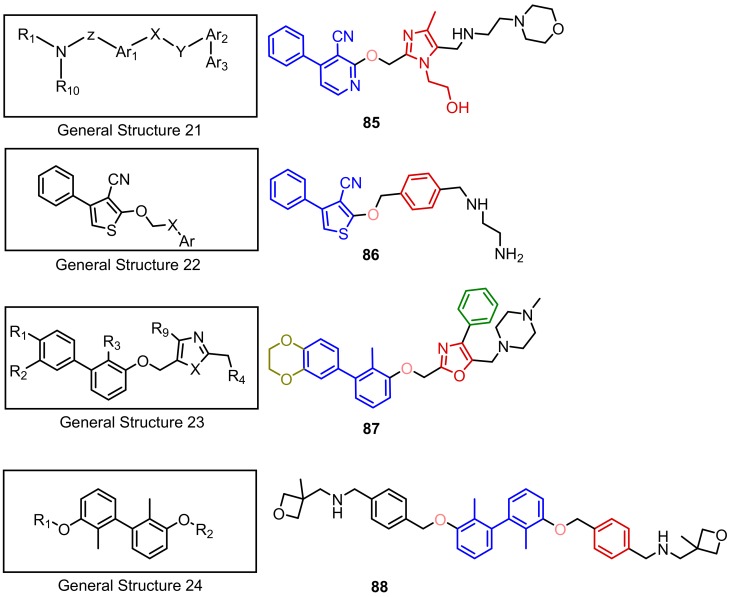
General structures 21–24 and examples of compounds **85**–**88** described by Dömling.

Dömling from the University of Groningen in the Netherlands disclosed a few novel scaffolds as inhibitors of the PD-1/PD-L1 axis. The activity of these compounds was verified by differential scanning fluorimetry (DSF) and NMR (General Structure 21), and additionally with MST (General structures 22–24). According to NMR binding assay the inhibitory activities of the disclosed compounds are in the range 0.001–1000 µM (IC_50_), but no precise value for a particular compound was provided (Figure 35) [91,92,93,94].

**Figure 36:**
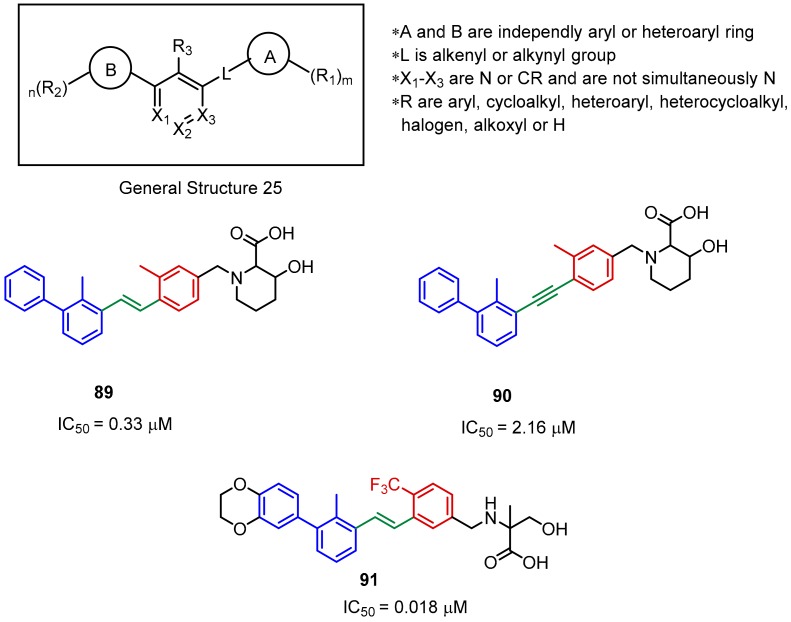
General structure and examples of the PD-1/PD-L1 inhibitors patented by the Maxinovel Corporation (**89**–**91**). Biphenyl fragments are shown in blue, phenyl fragments in red, the ethenyl and acetylenyl linkers in green.

At the beginning of 2018, Guangzhou Maxinovel Pharmaceuticals revealed a patent describing BMS-like immunomodulators. The main innovation, compared to BMS inhibitors, is the absence of the benzyl ether part, which is substituted by the ethenyl or ethynyl moieties (Figure 36). Albeit the significant differences in the geometry of the ethenyl or ethynyl linkers, both types of molecules inhibit the PD-1/PD-L1 interaction to a similar extent (Figure 36, **89** and **90**). Usually, the compounds that contain an acetylene fragment tend to be weaker PD-L1 binders. IC_50_ values from the HTRF experiments were in the range of 0.018 µM to over 10 µM [95].

During the American Association of Cancer Research (AACR) Annual Meeting in 2018, the Guangzhou Maxinovel Pharmaceuticals announced another small-molecule inhibitor named MAX-10129, declaring it has improved oral bioavailability as well as high tolerance in animal studies. The molecule indicated significant inhibition of tumor progression in the murine colorectal carcinoma MC-38 model. Additionally, MAX-10129 demonstrated anti-tumor efficacy in the combinations with an anti-CTLA4 antibody, an IDO inhibitor Epacadostat, a COX-2 inhibitor Celebrex, and the cytotoxic cisplatin. Triple and quadruple combined therapies that are based on this molecule are currently being tested; however, the exact structure of the announced compound remains unpublished [95,96].

## 3. Conclusions

Development of PD-1/PD-L1 inhibitors has become an exciting and growing research. Currently, there are six PD-1 or PD-L1 directed mAbs demonstrating anti-tumor efficacy to more than twenty cancer types. However, these therapies also have their weak sides coming mainly from the poor pharmacokinetic properties of mAbs. Therefore, the development of small molecules that could overcome these drawbacks could provide alternative or complementary therapies. A significant number of co-crystal structures published recently serves as a solid foundation for the rational design of the inhibitors targeting the PD-1/PD-L1 axis [5,41,42,43,44]. Moreover, the research so far has resulted in the discovery of several scaffolds that could be grouped into the macrocyclic peptides, peptides, oxadiazole or thiadiazole-derived peptidomimetics, and biaryl compounds.

**Figure 37:**
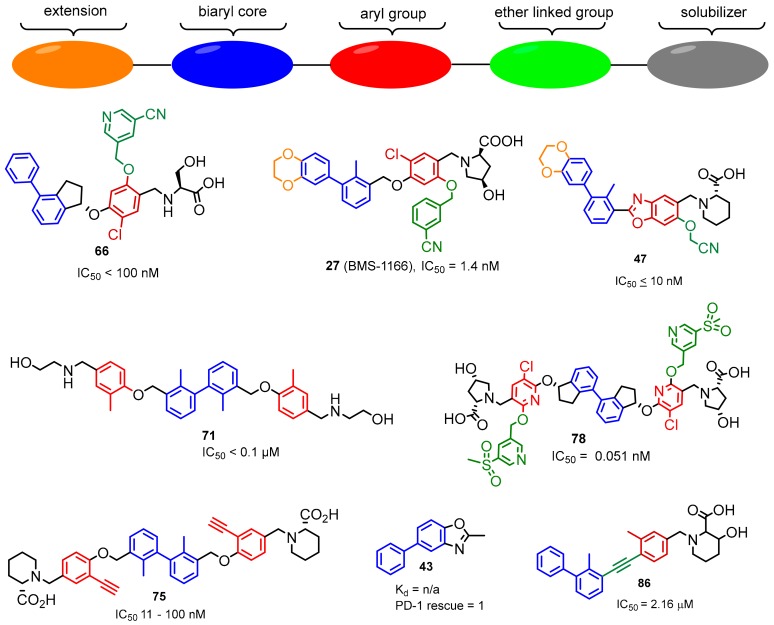
The general pattern in the development of PD-L1 inhibitors.

The biaryl compounds are the predominant class with up to sub nM binding potency towards PD-L1 (Figure 37). However in in vitro assays they are outperformed by the much more potent antibodies and macrocyclic peptides. Only the macrocyclic peptide 71 [40] was able to mimic about 37% of the PD-L1 antibody interactions, whereas the effect of BMS-1001 and BMS-1166 was significantly lower [43]. Interestingly, the data presented in [40] and [43] remain the only experimental comparison between organic compounds and commercially available antibodies so far. Nevertheless, one representative of this class of PD-1/PD-L1 inhibitors, MAX 10129, showed encouraging results in preclinical studies, e.g., anti-tumor efficacy in combination with an anti-CTLA4 antibody, and reportedly is undergoing further development towards clinical trials. Furthermore, the macrocyclic peptide BMS-986189 and the peptidomimetics CA-170 and CA-327 have shown promising results in phase I clinical trials.

Besides the difficulties of targeting the flat binding site of the protein–protein interaction between PD-1/PD-L1 and the early stage of the development of small-molecule PD-1/PD-L1 inhibitors, the attained results demonstrate a large amount of potential in this research area. The disclosure of the structures of CA-170, CA-327 and MAX-10129, and further academic and industrial developments, can lead to a new generation of highly potent orally available inhibitors, which can overcome some of the drawbacks of the current mAbs and improve immune checkpoint therapy by itself or in combination with other drugs.

## Supplementary Materials

The following are available online https://www.mdpi.com/1420-3049/24/11/2071/s1, Figure S1: The protein-protein interaction between the T-cell programed cell death protein-1 (PD-1) and the cancer cell borne programed cell death protein ligand-1 (PD-L1) keeps T cells from killing tumor cells in the body (left panel). Killing tumor cells by T cells can be accomplished by blocking the binding of PD-L1 to PD-1 with immune checkpoint inhibitors (ICIs) (anti-PD-L1 or anti-PD-1) (right panel)., Figure S2: The simplified concept of noncellular Assay Techniques described in the manuscript. (**A**) HTRF (Homogeneous Time Resolved Fluorescence) (**B**) ELISA (Enzyme-Linked Immunosorbent Assay) (**C**) AlphaLISATM human PD-1/PD-L1 binding assay (**D**) Amplified Luminescent Proximity Homogeneous Assay (ALPHA)., Figure S3: The concept of the cellular Assay Techniques described in the manuscript. (**A**) INFγ-release assay (**B**) The PD1/PDL1 Blockade Bioassay (**C**) The T-cell proliferation assay., Table S1: The list of abbreviations used in the manuscript together with short explanation of biological terms., Table S2: Structures and references to source materials of compound mentioned in the main text.
