# Supplementary material for: Development of the Inhibitors That Target the PD-1/PD-L1 Interaction—A Brief Look at Progress on Small Molecules, Peptides and Macrocycles
Source: Molecules. 2019 May 30;24(11):2071. doi: 10.3390/molecules24112071 (PMC6600339; doi:10.3390/molecules24112071)
Supplement: Supplementary file 1 [file molecules-24-02071-s001.pdf]

Supplementary material for

Development of the inhibitors that target the PD-1/PD-L1 interaction – a brief look at progress on small molecules, peptides and macrocycles

Katarzyna A. Guzik[1,*], Marcin Tomala[1], Damian Muszak[1], Magdalena Konieczny[1], Aleksandra Hec[2], Urszula Błaszkiewicz[2], Marcin Pustuła[1], Roberto Butera[3], Alexander Dömling[3] and Tad A. Holak[1]

1 Faculty of Chemistry, Jagiellonian University, Gronostajowa 2, 30-387 Krakow, Poland
2 Recepton sp. z o.o, Michala Bobrzynskiego 14, 30-348 Krakow, Poland
3 Department for Drug Design, University of Groningen, A. Deusinglaan 9, AV 9713 Groningen, the Netherlands
* Correspondence: katarzyna.guzik01@gmail.com

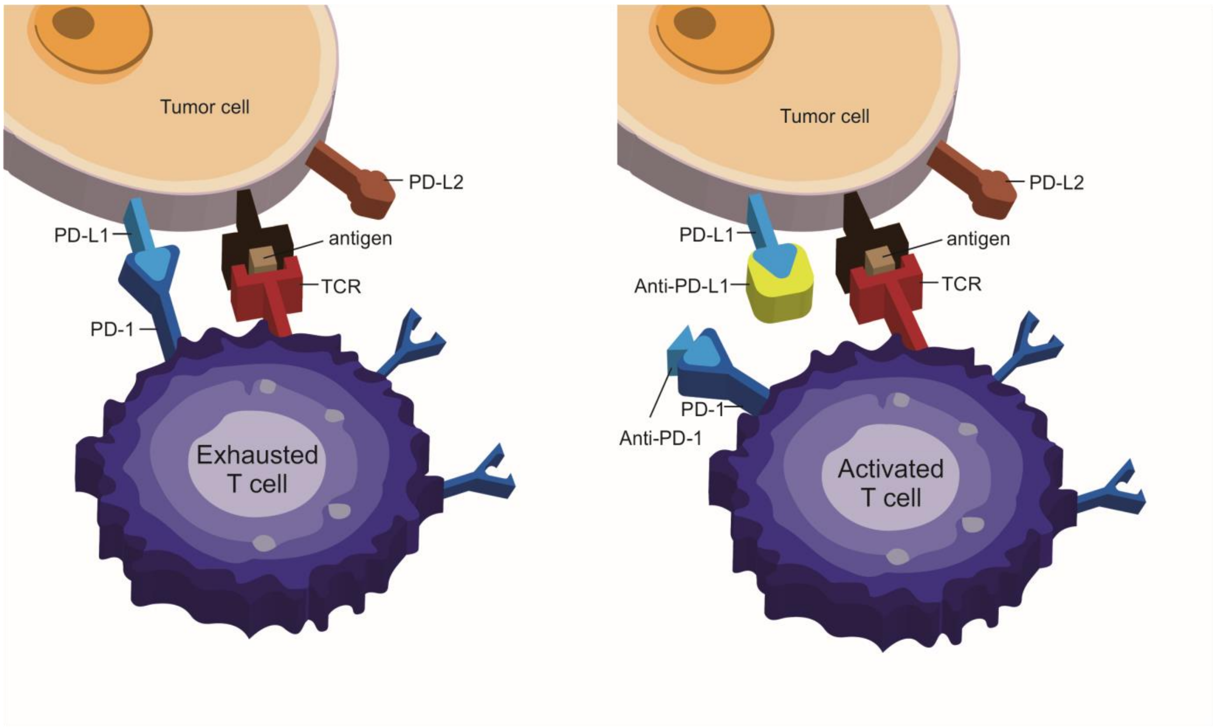

Figure S1. The protein-protein interaction between the T-cell programed cell death protein-1 (PD-1) and the cancer cell borne programed cell death protein ligand-1 (PD-L1) keeps T cells from killing tumor cells in the body (left panel). Killing tumor cells by T cells can be accomplished by blocking the binding of PD-L1 to PD-1 with immune checkpoint inhibitors (ICIs) (anti-PD-L1 or anti-PD-1)(right panel).

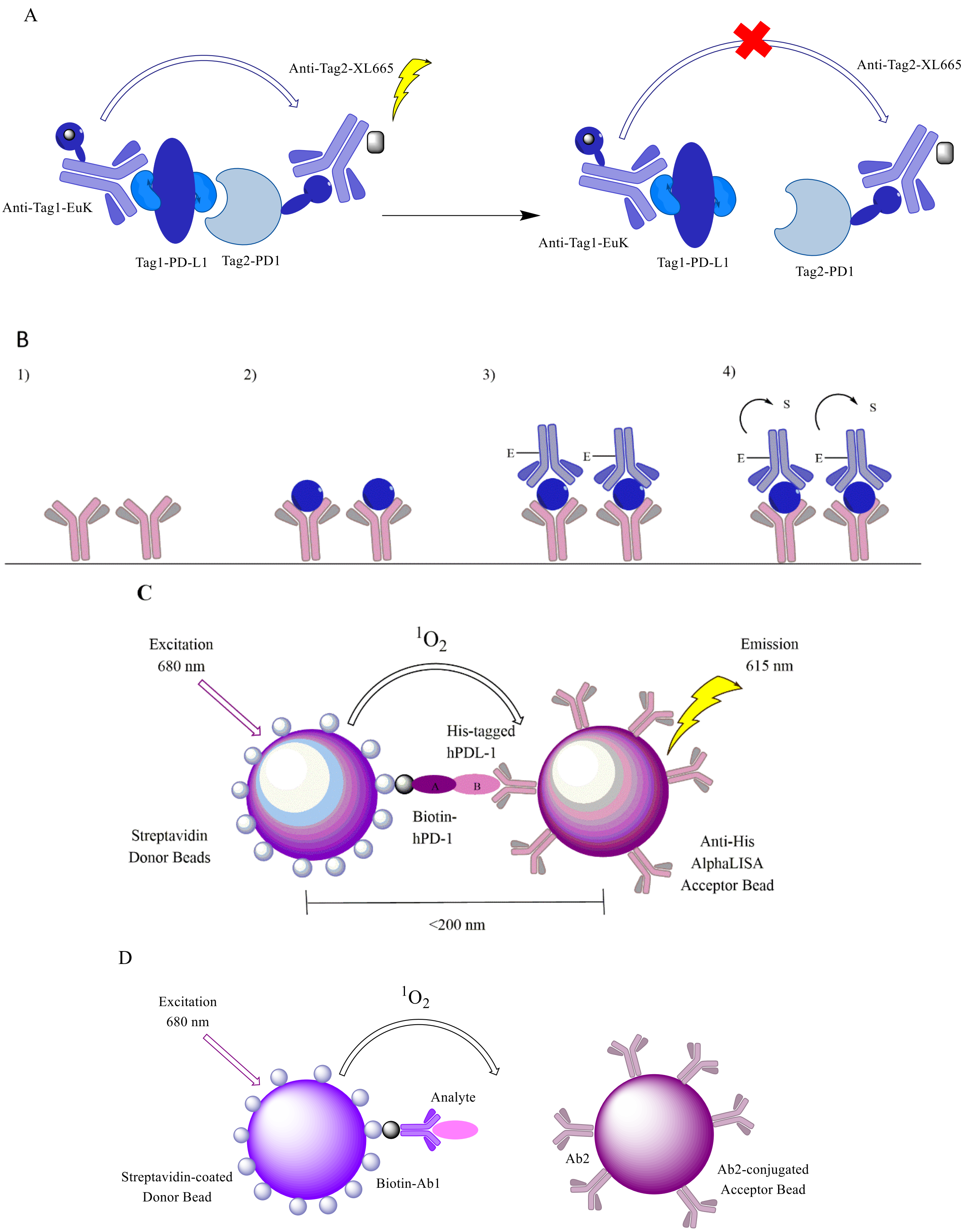

Figure S2: The simplified concept of noncellular Assay Techniques described in the manuscript. A) HTRF (Homogeneous Time Resolved Fluorescence) B) ELISA (Enzyme-Linked Immunosorbent Assay) C) AlphaLISATM human PD-1/PD-L1 binding assay D) Amplified Luminescent Proximity Homogeneous Assay (ALPHA)

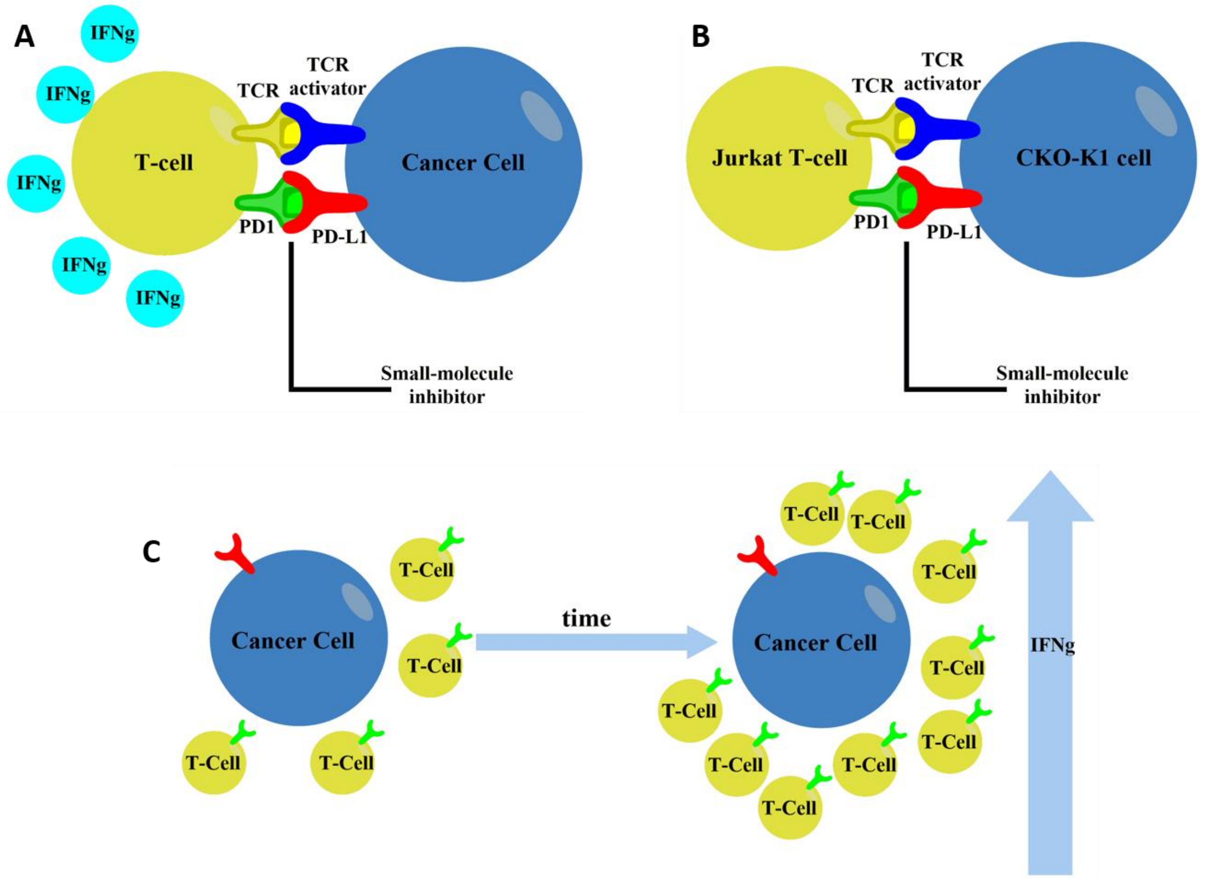

Figure S3: The concept of the cellular Assay Techniques described in the manuscript. A) INFγ-release assay B) The PD1/PDL1 Blockade Bioassay C) The T-cell proliferation assay.

Table S1: The list of abbreviations used in the manuscript together with short explanation of biological terms.

Abbreviation	Explanation
PD-1 (CD279)	Programmed cell death receptor 1, protein expressed on the surface of T cells, B cells and macrophages regulating the immune system's response by downregulation and self-tolerance promotion.
PD-L1 (B7-H1, CD274)	Programmed cell death-ligand 1, transmembrane protein binding to PD-1, supressing immune system, found on activated T cells, B cells, myeloid cells and cancer cells.
PD-L2 (B7-DC, CD273)	Programmed cell death-ligand 2, transmembrane protein binding to PD-1, supressing immune system, found on activated T cells, B cells, myeloid cells and cancer cells.
PD-1/PD-L1	The interaction of programmed cell death receptor 1 with its ligand PD-L1.
Anti-PD-L1	Factor (antibody, protein, macrocycle or small molecule) inhibiting the activity of PD-L1.
Ig	Immunoglobulin domain. This type of protein domain consists of a seven to nine antiparallel β-strands forming a barrel like shape.
TNF/TNFR	Tumor necrosis factor. The superfamily of transmembrane

	proteins expressed by immune cells which can be released from cell membrane and function as cytokine. These proteins regulate mainly immune response and inflammation.
IgV domain	Immunoglobulin variable domain. The immunoglobulin domain consisting of 9 beta strands
IgC domain	Immunoglobulin variable domain. Immunoglobulin domain consisting of 7 beta strands
ITSM	Immunoreceptor tyrosine-based switch motif. Conserved sequence of amino acids found in the cytoplasmic region of receptors of the immune system. An ITSM can activate or inhibit signal, depending on the type of immune cell, the receptor and the bound protein.
ITIM	Immunoreceptor tyrosine-based inhibition motif. A conserved sequence of amino acids found in the cytoplasmic tails of the receptors of the immune system. The ITIM conveys to signal inhibition.
CTLA-4	Cytotoxic T-lymphocyte-associated protein 4. An immune checkpoint protein receptor expressed in regulatory T cells, downregulating immune response.
CD-28	Cluster of differentiation 28. Protein expressed on T cells; the receptor provides co-stimulatory signals contributed to T cell activation and survival.
CD-80	Cluster of differentiation 80. Protein found on B cells, monocytes and dendritic cells, contributes to a costimulatory signalling, leading to T cell activation.
APCs	Antigen-presenting cells. Cells presenting on their surfaces complexed antigens. T cells are able to recognize these complex using T cell receptor (TCR).
K_D	Dissociation constant.
TCR	T-cell receptor. The protein is expressed on the surface of T cells, allows recognition of antigen fragments.
FDA	Food and Drug Administration
mAbs	Monoclonal antibodies produced by identical immune cells.
SAR	Structure–activity relationship, relationship between the 3D structure of a molecule and its biological activity.
EC_{50}	The half maximal effective concentration is the concentration of a drug which induces the half of the maximal response.
INF-γ	Interferon gamma, a soluble cytokine that is an activator of macrophages, secreted mainly by T cells.
CLT assay	A chemiluminescent test; based on a luminescence measurement.
MSPA assay	Murine splenocyte proliferation assay
PBMC assay	Human peripheral blood mononuclear cell proliferation assay
IC_{50}	The half maximal inhibitory concentration, the concentration of a drug which inhibits biological process by half.
NMR-titration	Nuclear magnetic resonance titration, biophysical method based on the observation of changes in chemical shifts of

	the protein signals upon the addition of tested compound.
DSF	Differential scanning fluorimetry, technique based on the monitoring of thermally induced protein denaturation, detected as changes in fluorescence of the dye that binds to unfolded protein. Tested molecule may stabilize or destabilize protein what is observed as changes in the denaturation temperature.
VISTA	V-domain Ig suppressor of T cell activation, transmembrane protein expressed on hematopoietic cells, especially myeloid and granulocytes. Low expression was proven on T cells also. VISTA exhibit both receptor and ligand functions and supresses activation of T cells.
TIM-3	T cell immunoglobulin and mucin-domain containing-3, protein expressed on cytotoxic T cells , regulatory T cells, dendritic cells, NK cells and monocytes. Overexpression was observed in lymphocytes in various types of cancer. TIM-3 mediates cytotoxic cells exhaustion and regulates macrophages activation.
INFγ-release assay	Interferon gamma release assay, the method based on monitoring of Inf-γ concentration during anti-PD-1/PD-L1 molecule treatment. Inhibition of the complex formation is positively correlated with amount of released INF-γ.
HTRF assay	Homogeneous time resolved fluorescence assay, biophysical method based on fluorescence measurements. The fluorescence is possible by FRET effect when two proteins are in close proximity (<8-9 nm); the presence of binding agents causing a dissociation of protein complex results in the loss of fluorescence.
AIDA-NMR	Antagonist induced dissociation assay, a type of competition binding NMR experiment based on the observation of protein signals disappearing upon binding to a large protein fragment. Used for validation of inhibitor action on protein-protein interactions.
ELISA	Enzyme-linked immunosorbent assay. A solid-phase immunoassay allowing detecting a ligand using an antibody against that target.
MLR assay	Mixed lymphocyte reaction assay. Test used to check the safety of a drug, commonly used as a part of FDA procedures. Basically, it measures the reaction of lymphocytes to tested substance.
ALPHA	Amplified luminescent proximity homogeneous assay, method based on photochemical interaction of two proteins. The singlet-oxygen-mediated interaction is only possible when proteins are in close proximity and therefore decreases when the complex is dissociated by a tested binding agent.
AlphaLISA human PD-1/PD-L1 binding assay	A variation of the AlphaLISA assay with a shortened protocol.

Table S2: Structures and references to source materials of compound mentioned in the main text.

Symbol	Structure	Reference
1	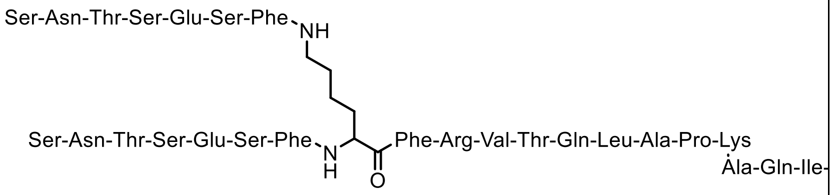 **1**	36. Sasikumar, P.G.; Satyam, L.K.; Shrimali, R.K.; Subbarao, K.; Ramachandra, R.; Vadlamani, S.; Reddy, A.; Kumar, A.; Srinivas, A.; Reddy, S.; et al. Abstract 2850: Demonstration of anti-tumor efficacy in multiple preclinical cancer models using a novel peptide inhibitor (Aurigene-012) of the PD1 signaling pathway. *Cancer Res.* **2012**, *72*, 2850 LP – 2850.
2	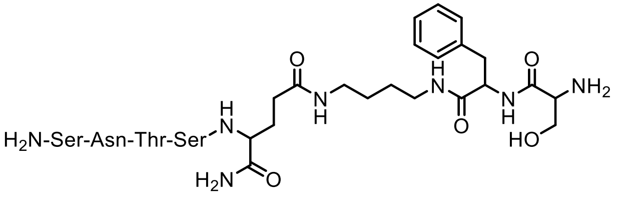 **2**	37. Sasikumar, P.G.N.; Ramachandra, M.; Vadlamani, S.K.; Shrimali, K.; Subbarao, K. Therapeutic Compounds for Immunomodulation. WO2012168944, December 13, 2012.
3	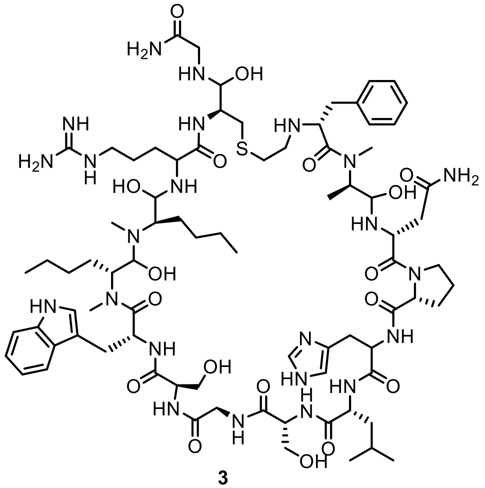 **3**	40. Magiera-Mularz, K.; Skalniak, L.; Zak, K.M.; Musielak, B.; Rudzinska-Szostak, E.; Berlicki, Ł.; Kocik, J.; Grudnik, P.; Sala, D.; Zarganes-Tzitzikas, T.; et al. Bioactive Macrocyclic Inhibitors of the PD-1/PD-L1 Immune Checkpoint. *Angew. Chem. Int. Ed. Engl.* **2017**, *56*, 13732–13735.

4	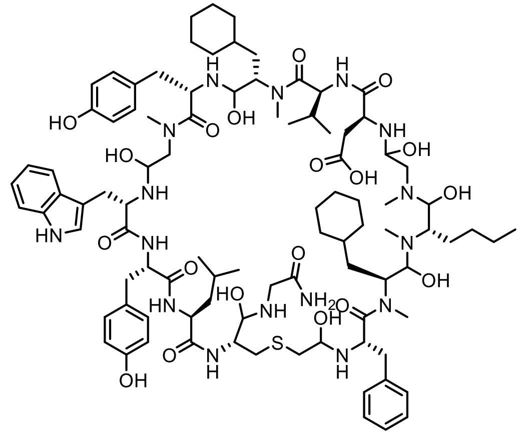 **4**	40. Magiera-Mularz, K.; Skalniak, L.; Zak, K.M.; Musielak, B.; Rudzinska-Szostak, E.; Berlicki, Ł.; Kocik, J.; Grudnik, P.; Sala, D.; Zarganes-Tzitzikas, T.; et al. Bioactive Macrocyclic Inhibitors of the PD-1/PD-L1 Immune Checkpoint. *Angew. Chem. Int. Ed. Engl.* **2017**, *56*, 13732–13735.
5	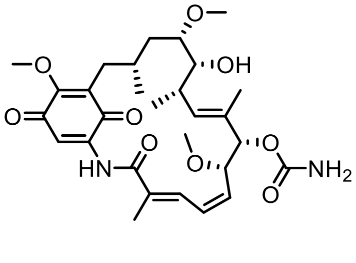 **5**	45. Patil, S.P.; Yoon, S.-C.; Aradhya, A.G.; Hofer, J.; Fink, M.A.; Enley, E.S.; Fisher, J.E.; Herb, M.C.; Klingos, A.; Proulx, J.T.; et al. Macrocyclic Compounds from Ansamycin Antibiotic Class as Inhibitors of PD1–PDL1 Protein–Protein Interaction. *Chem. Pharm. Bull.* **2018**, *66*, 773–778.
6	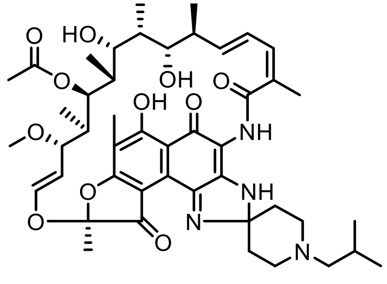 **6**	

7	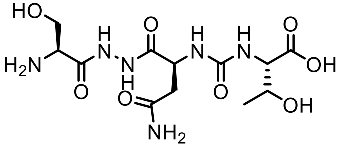 **7**	46. Sasikumar, P.G.N.; Ramachandra, M.; Naremaddepalli, S.S.S. Peptidomimetic Compounds as Immunomdulators. US20130237580, September 12, 2013.
8	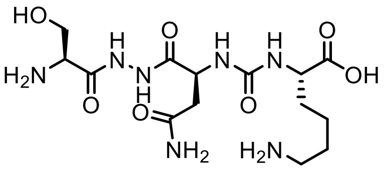 **8**	28. Weinmann, H. Cancer Immunotherapy: Selected Targets and Small-Molecule Modulators. *ChemMedChem* **2016**, *11*, 450–466.<br>34. Chen, T.; Li, Q.; Liu, Z.; Chen, Y.; Feng, F.; Sun, H. Peptide-based and small synthetic molecule inhibitors on PD-1/PD-L1 pathway: A new choice for immunotherapy? *Eur. J. Med. Chem.* **2019**, *161*, 378–398.<br>47. Sasikumar P. G. N.; Ramachandra M.; Naremaddepalli S. S. S. Immunomodulating peptidomimetic derivatives. WO2015036927A1, March 19, 2015.<br>48. Sasikumar P. G. N.; Ramachandra M.; Naremaddepalli S. S. S. Therapeutic immunomodulating compounds. WO2015044900A1, April 2, 2015.
9	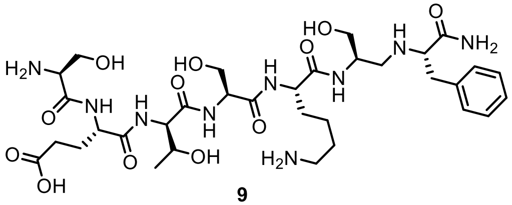 **9**	28. Weinmann, H. Cancer Immunotherapy: Selected Targets and Small-Molecule Modulators. *ChemMedChem* **2016**, *11*, 450–466.<br>33. Wang, T.; Wu, X.; Guo, C.; Zhang, K.; Xu, J.; Li, Z.; Jiang, S. Development of Inhibitors of the Programmed Cell Death-1/Programmed Cell Death-Ligand 1 Signaling Pathway. *J. Med. Chem.* **2019**, *62*, 1715–1730.<br>48. Sasikumar P. G. N.; Ramachandra M.; Naremaddepalli S. S. S. Therapeutic immunomodulating compounds. WO2015044900A1, April 2, 2015.
10	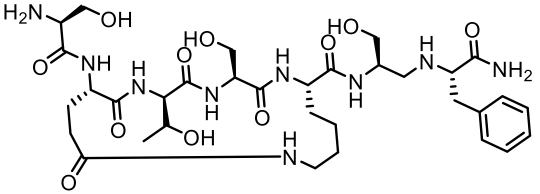 **10**	
11	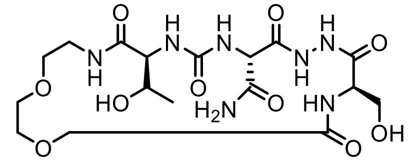 **11**	

12	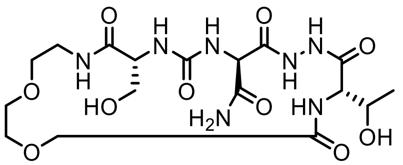<br>**12**	28. Weinmann, H. Cancer Immunotherapy: Selected Targets and Small-Molecule Modulators. *ChemMedChem* **2016**, *11*, 450–466.<br>33. Wang, T.; Wu, X.; Guo, C.; Zhang, K.; Xu, J.; Li, Z.; Jiang, S. Development of Inhibitors of the Programmed Cell Death-1/Programmed Cell Death-Ligand 1 Signaling Pathway. *J. Med. Chem.* **2019**, *62*, 1715–1730.<br>48. Sasikumar P. G. N.; Ramachandra M.; Naremaddepalli S. S. S. Therapeutic immunomodulating compounds. WO2015044900A1, April 2, 2015.
13	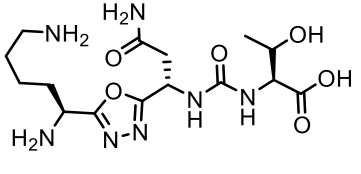<br>**13**	53. Sasikumar, P.G.N.; Ramachandra, M.; Naremaddepalli, S.S.S. Cyclic substituted-1,3,4-oxadiazole and thiadiazole compounds as immunomodulators. WO2018051255A1, March 22, 2018.<br>54. Sasikumar, P.G.N.; Ramachandra, M.; Naremaddepalli, S.S.S. Cyclic substituted-1,2,4-oxadiazole and thiadiazole compounds as immunomodulators. WO2018051254A1, March 22, 2018.
14	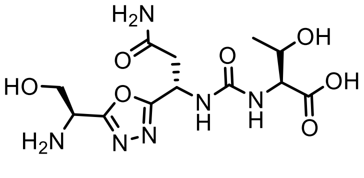<br>**14**	
15	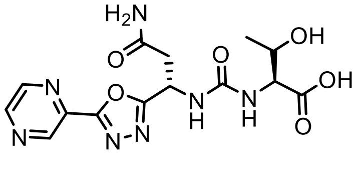<br>**15**	55. Sasikumar, P.G.N.; Ramachandra, M.; Naremaddepalli, S.S.S.; Prasad, A. 3-Substituted 1,3,4-oxadiazole and thiadiazole compounds as immunomodulators. WO2016142894A1, September 15, 2016.<br>56. Sasikumar, P.G.N.; Ramachandra, M.; Naremaddepalli, S.S.S. 1,2,4-oxadiazole derivatives as immunomodulators. US10173989B2, January 8, 2019.
16	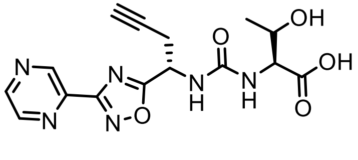<br>**16**	

17	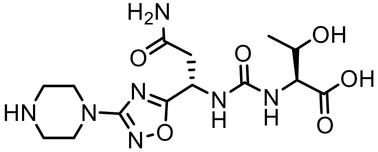 **17**	55. Sasikumar, P.G.N.; Ramachandra, M.; Naremaddepalli, S.S.S.; Prasad, A. 3-Substituted 1,3,4-oxadiazole and thiadiazole compounds as immunomodulators. WO2016142894A1, September 15, 2016.<br>56. Sasikumar, P.G.N.; Ramachandra, M.; Naremaddepalli, S.S.S. 1,2,4-oxadiazole derivatives as immunomodulators. US10173989B2, January 8, 2019.
18	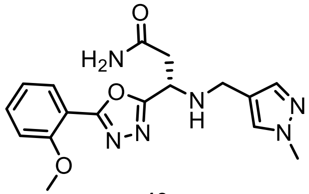 **18**	56. Sasikumar, P.G.N.; Ramachandra, M.; Naremaddepalli, S.S.S. 1,2,4-oxadiazole derivatives as immunomodulators. US10173989B2, January 8, 2019.<br>57. Sasikumar, P.G.N.; Ramachandra, M.; Naremaddepalli, S.S.S. 1,3,4-oxadiazole and 1,3,4-thiadiazole derivatives as immunomodulators. US10160736B2, December 25, 2018.
19	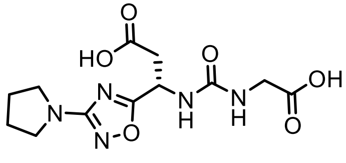 **19**	
20	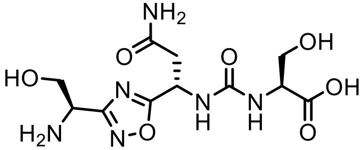 **20**	
21	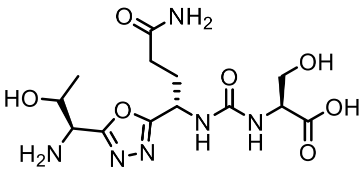 **21**	

22	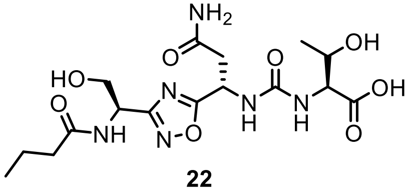<br>**22**	56. Sasikumar, P.G.N.; Ramachandra, M.; Naremaddepalli, S.S.S. 1,2,4-oxadiazole derivatives as immunomodulators. US10173989B2, January 8, 2019.<br>57. Sasikumar, P.G.N.; Ramachandra, M.; Naremaddepalli, S.S.S. 1,3,4-oxadiazole and 1,3,4-thiadiazole derivatives as immunomodulators. US10160736B2, December 25, 2018.
23	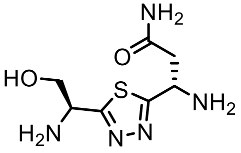<br>**23**	
24	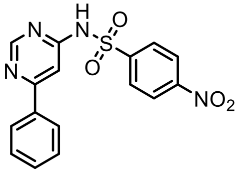<br>**24**	59. Sharpe, A.H.; Butte, M.J.; Oyama, S. 2011, Modulators of immunoinhibitory receptor PD-1, and methods of use thereof, 7 July 2011. WO2011082400 A2
25	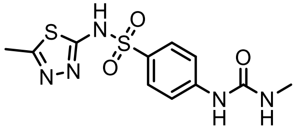<br>**25**	
26 (BMS-202)	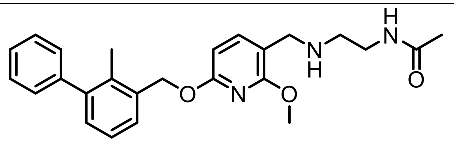<br>**26** (BMS-202)	60. Chupak L.S.; Zheng X. Compounds useful as immunomodulators. WO2015034820 A1, March 12, 2015.<br>61. Chupak, L. S.; Ding, M.; Martin, S. W.; Zheng, X.; Hewawasam, P.; Connolly, T. P.; Xu, N.; Yeung, K.-S.; Zhu, J.; Langley, D. R.; Tenney, D. J.; Scola, P. M.; Mingo, P. A. Compounds Useful as Immunomodulators. WO2015160641, October 22, 2015.<br>62. Yeung, K. S.; Connolly, T. P.; Frennesson, D. B.; Grant-Young, K. A.; Hewawasam, P.;

26a<br>(BMS-1165)	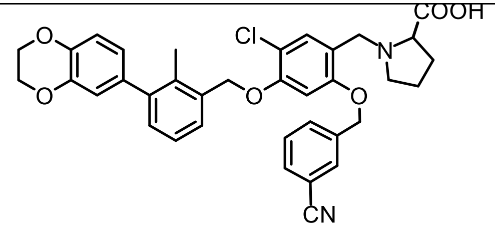<br>**26a** (BMS-1165)	Langley, D. R.; Meng, Z.; Mull E.; Parcella, K. E.; Saulnier, M. G.; Sun; L. Q.; Wang; A. X.; Xu, N.; Zhu, J.; Scola, P. M. Compounds Useful as Immunomodulators. WO2017/066227, April 20, 2017.<br>63. Yeung, K. S.; Grant-Young, K. A.; Zhu J.; Saulnier, M. G.; Frennesson, D. B.; Meng, Z.; Scola, P. M.; 1,3-dihydroxy-phenyl derivatives useful as immunomodulators. WO2018/009505 A1, January 11, 2018.<br>64. Yeung, K. S.; Grant-Young, K. A.; Zhu J.; Saulnier, M. G.; Frennesson, D. B.; Langley, D. R.; Hewawasam, P.; Wang; A. X.; Zhang, Z.; Meng, Z.; Sun; L. Q.; Mull, E.; Scola, P. M. Biaryl Compounds Useful as Immunomodulators.WO2018/044963A1, March 8, 2018.<br>65. Yeung, K. S.; St. Laurent, D. R.; Romine, J. L.; Scola, P. M.; Substituted isoquionline derivatives as immunomodulators. WO2018/183171 A1, October 18, 2018.
26b<br>(BMS-1016)	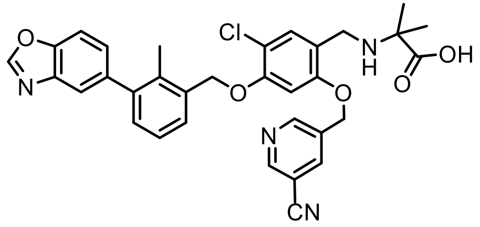<br>**26b** (BMS-1016)	
27<br>(BMS-2007)	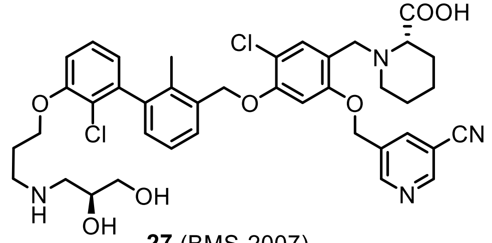<br>**27** (BMS-2007)	
28<br>(BMS-40210)	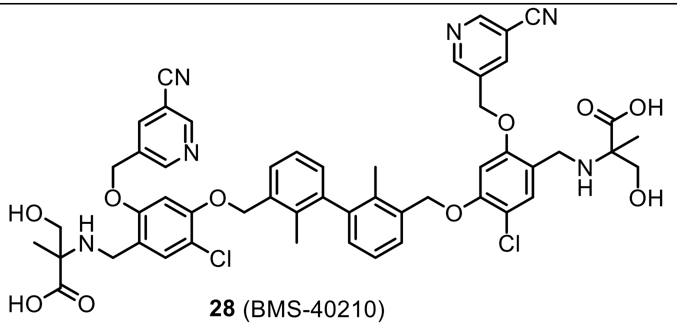<br>**28** (BMS-40210)	

29<br>(BMS-1001)	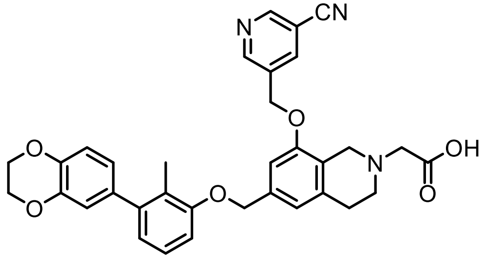<br>**29** (BMS-1001)	
30<br>(BMS-8)	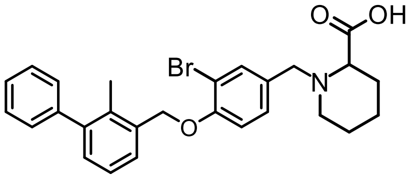<br>**30** (BMS-8)	41. Zak, K.M.; Grudnik, P.; Guzik, K.; Zieba, B.J.; Musielak, B.; Dömling, A.; Dubin, G.; Holak, T.A. Structural basis for small molecule targeting of the programmed death ligand 1 (PD-L1). *Oncotarget* **2016**, *7*, 30323–30335.<br>43. Skalniak, L.; Zak, K. M.; Guzik, K.; Magiera, K.; Musielak, B.; Pachota, M.; Szelazek, B.; Kocik, J.; Grudnik. P.; Tomala, M.; Krzanik, S.; Pyrc, K.; Dömling, A.; Dubin, G.; Holak, T. A. Small-molecule inhibitors of PD-1/PD-L1 immune checkpoint alleviate the PD-L1-induced exhaustion of T-cells. *Oncotarget*, **2017**, *8*(42), 72167–72181.<br>44. Perry, E.; Mills, J.J.; Zhao, B.; Wang, F.; Sun, Q.; Christov, P.P.; Tarr, J.C.; Rietz, T.A.; Olejniczak, E.T.; Lee, T.; et al. Fragment-based screening of programmed death ligand 1 (PD-L1). *Bioorg. Med. Chem. Lett.* **2019**, *29*, 786–790.
31<br>(BMS-37)	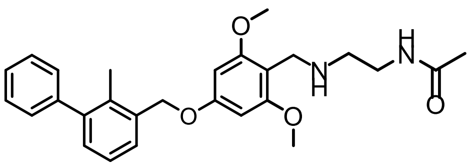<br>**31** (BMS-37)	
32<br>(BMS-200)	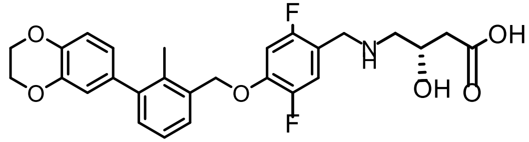<br>**32** (BMS-200)	

33 (BMS-1166)	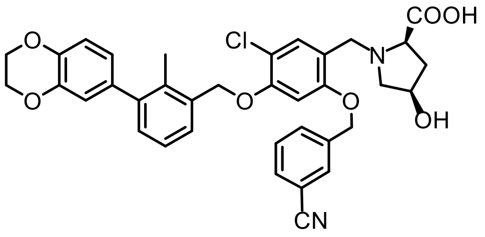 **33** (BMS-1166)	
34 (BMS-1001)	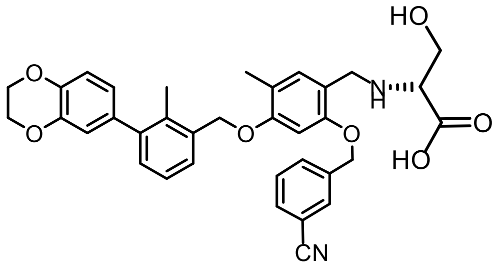 **34** (BMS-1001)	
35 (BMS-105)	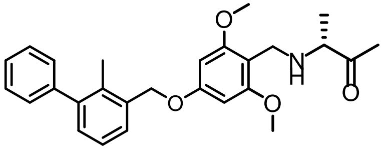 **35** (BMS-105)	
36	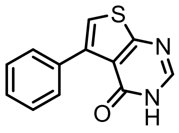 **36**	44. Perry, E.; Mills, J.J.; Zhao, B.; Wang, F.; Sun, Q.; Christov, P.P.; Tarr, J.C.; Rietz, T.A.; Olejniczak, E.T.; Lee, T.; et al. Fragment-based screening of programmed death ligand 1 (PD-L1). *Bioorg. Med. Chem. Lett.* **2019**, *29*, 786–790.

37	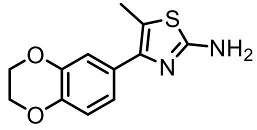 **37**	
38	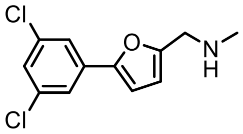 **38**	
39	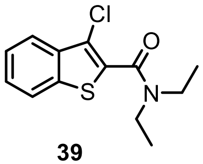 **39**	
40	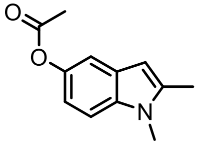 **40**	
41	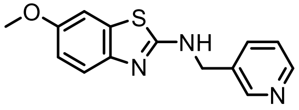 **41**	
42	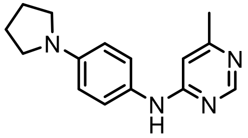 **42**	

43	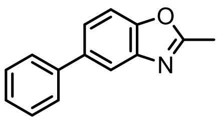 **43**	
44	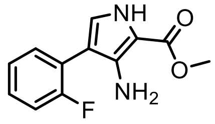 **44**	
45	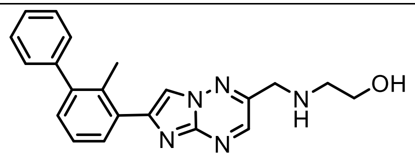 **45**	69. Wu, L.; Shen, B.; Li, J.; Li, Z.; Liu, K.; Zhang, F.; Yao, W. 2017, Heterocyclic compounds as immunomodulators, 20 April 2017. US20170107216 A1
46	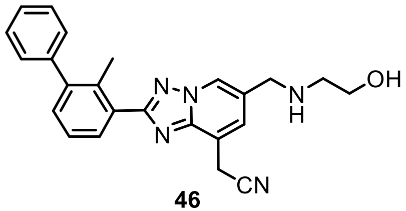 **46**	
47	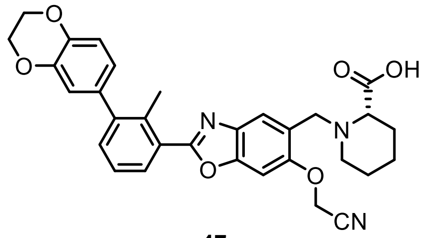 **47**	70. Li, J.; Wu, L.; Yao, W. 2017, Heterocyclic compounds as immunomodulators, 26 May 2017. WO2017087777 A1

48	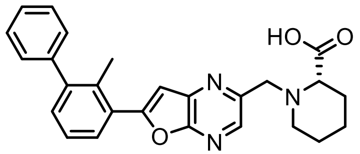 **48**	
49	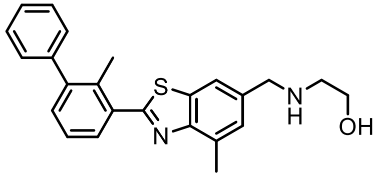 **49**	
50	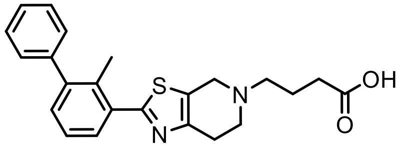 **50**	71. Li, Z; Wu, L.; Yao, W. 2017, Heterocyclic compounds as immunomodulators, 09 November 2017. WO2017192961 A1
51	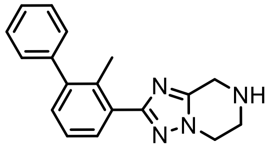 **51**	
52	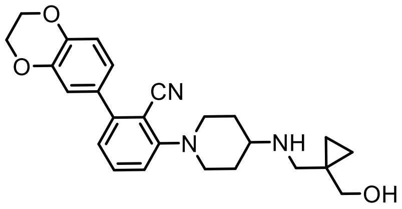 **52**	

53	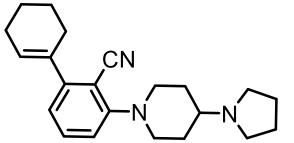 **53**	
54	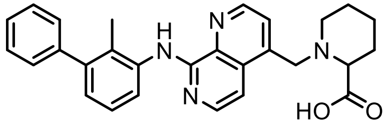 **54**	73. Lajkiewicz, N.; Wu, L.; Yao, W. 2017, Heterocyclic compounds as immunomodulators, 22 June 2017. US 20170174679 A1
55	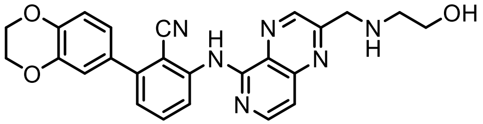 **55**	
56	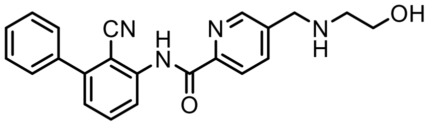 **56**	74. Wu, L.; Yu, Z.; Zhang, F.; Yao, W. 2017, *N*-phenyl-pyridine-2-carboxamide derivatives and their use as PD-1/PD-L1 protein/protein interaction modulators, 22 June 2017. WO2017106634 A1
57	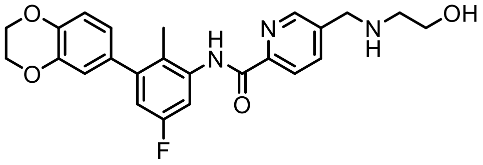 **57**	

58	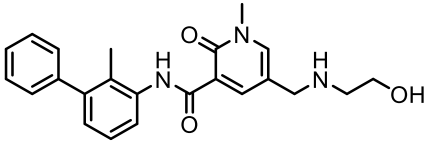<br>**58**	75. Yu, Z.; Wu, L.; Yao, W. 2018, Heterocyclic compounds as immunomodulators, 18 January 2018. WO2018013789 A1
59	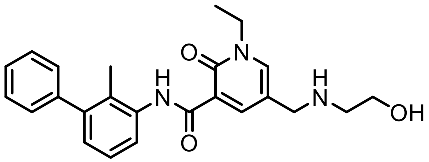<br>**59**	
60	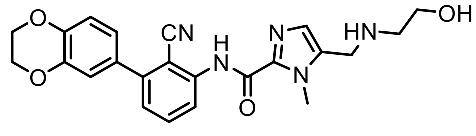<br>**60**	76. Wu, L.; Zhang, F.; Yao, W. 2018, Heterocyclic compounds as immunomodulators, 08 March 2018. WO2018044783 A1<br>77. Xiao, K.; Zhang, F.; Wu, L.; Yao, W. 2017, Heterocyclic compounds as immunomodulators, 21 December 2017. US20170362253 A1
61	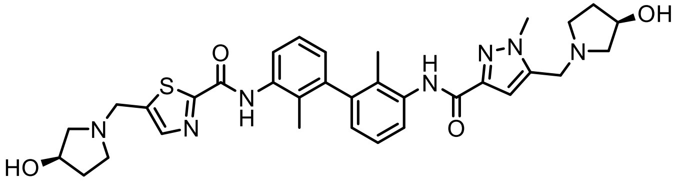<br>**61**	
62	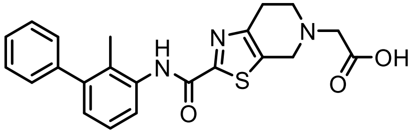<br>**62**	

63	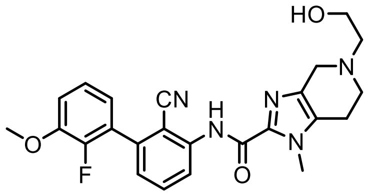 **63**	
64	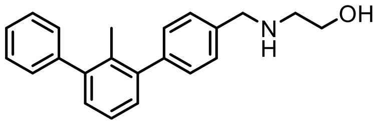 **64**	78. Wu, L.; Qian D.Q.; Lu, L.; Lajkiewicz, N., Konkol, L.C.; Li, Z.; Zhang, F.; Li, J.; Wang, H.; Xu, M.; Xiao, K.; Yao, W. 2018b, Heterocyclic compounds as immunomodulators, 28 June 2018. US 20180177784 A1
65	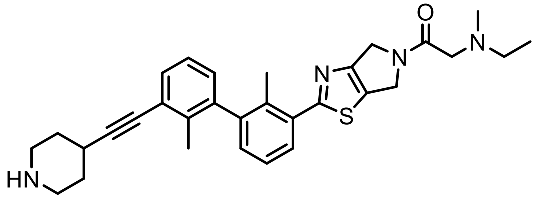 **65**	
66	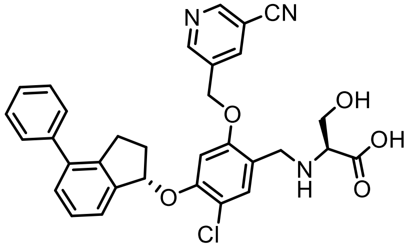 **66**	79. Lange, C.; McMurtrie D.J; Malathong, V.; Punna, S.; Singh, R.; Yang, J.; Zhang, P. 2018, Immunomodulator Compounds, 11 January 2018, WO2018005374A1.<br>80. Lange, C.; McMurtrie, D.J.; Malathong, V.; Mali, V.R.; Mcmahon, J.; Roth, H.S.; Singh, R.; Wang, Y.; Yang, J.; Zhang, P. 2018, Immunomodulator Compounds, 31 January 2019, WO2019023575A1<br>81. Vilalta Colomer, M.; Li, S.; Malathong, V.; Lange, C.; McMurtrie, D.; Yang, J.; Roth, H.; McMahon, J.; Campbell, J.J.; Ertl, L.S.; Ong, R.; Wang, Y.; Zhao, N.; Yau, S.; Dang, T.; Zhang, P.; Schall, T.J.; Singh, R.; Punna, S., A small molecule human PD-1/PD-L1 inhibitor promotes T cell immune activation and reduces tumor growth in a preclinical model,

67	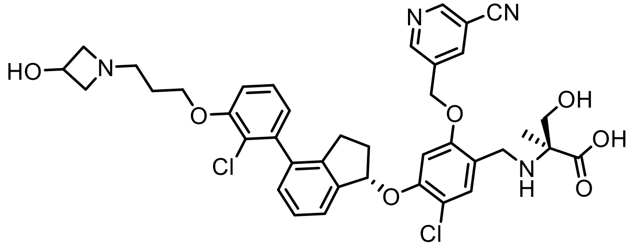 **67**	*Annals of Oncology* **2018** *29* (suppl_10): x24-x38. 10.1093/annonc/mdy487.001
68	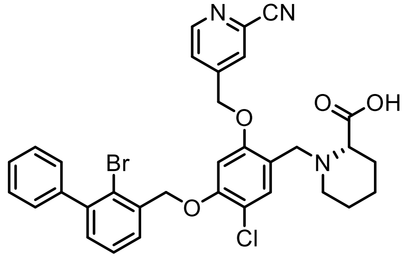 **68**	82. Feng, Z.; Chen, X.; Yang, Y.; Zhou, C.; Lai, F.; Ji, M.; Jing, X.; Xue, N.; Zheng, Y; Chen, H.; Li, L. Phenylate Derivative, Preparation Method Therefor, and Pharmaceutical Composition and Uses Therof, 30 November 2017. WO2017202276 A1
69	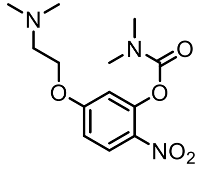 **69**	83. Li, S.; Xiao, J.; Liu, A.; Wei, X.; Zhong, W.; Zheng, Z.; Wang, X.; Xie, Y.; Zhao, G.; Li, H. Resorcinol Compound and Medicinal Use Thereof. CN107286057 A, 24 October 2017
70	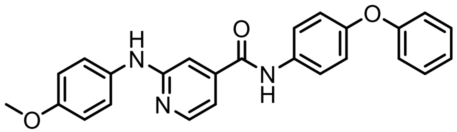 **70**	84. Sun, H.; Xin, T.; Wen, X.; Wu, Y.; Yuan, H. 2-Substituted Isonicotinic Acid Compound, Preparation Method and Application Thereof. CN106632021, 10 May 2017.

71	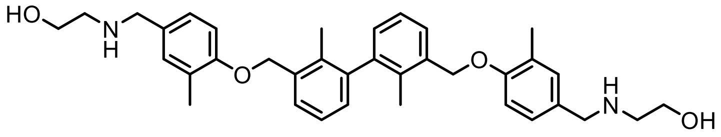 **71**	85. Wang, M. Symmetric or Semi-symmetric Compounds Useful As Immunomodulators, 08 February 2019, WO2018/026971
72	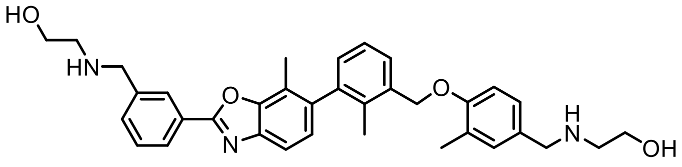 **72**	
73	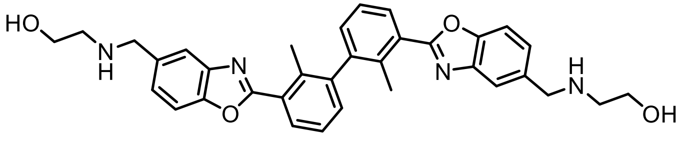 **73**	
74	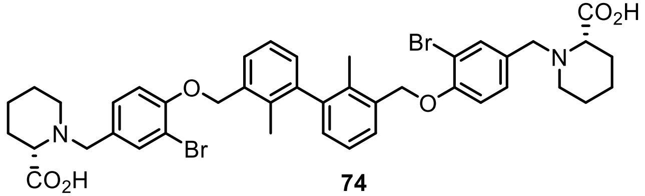 **74**	86. Webber, S.; Almassy, R. J. Immune Checkpoint Inhibitors Compositions and Methods Thereof. WO2018/045142 A1, March 8, 2018
75	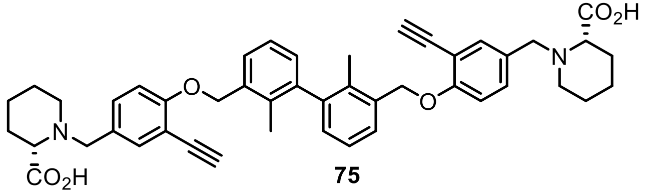 **75**	

76	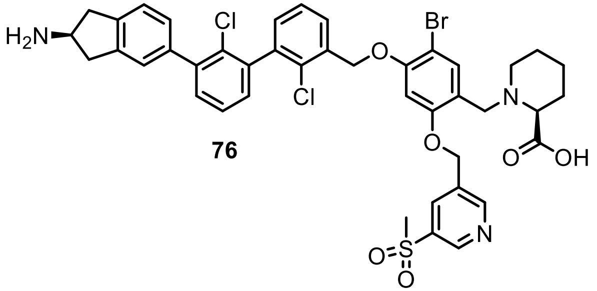 **76**	87. Aktoudianakis, E.; Appleby, T.; Aesop, C.; Zhimin, D.; Graupe, M.; Guerrero, J.; Jabri, S.; Lad, L.; Machicao Tello, P.A.; Medley, J.W.; Metobo, S.E.; Mukherjee, P.K.; Naduthambi, D.; Notte, G.; Parkhill, E.Q.; Phillips, B.W.; Simonovich, S.P.; Squires, N.H.; Venkataramani, C.; Wang, P.S.; Watkins, W.J.; Xu, J.; Yang, K.S.; Ziebenhaus, C.A. PD-1/PD-L1 INHIBITORS, 25 October 2018, US 2018/0305315 A1
77	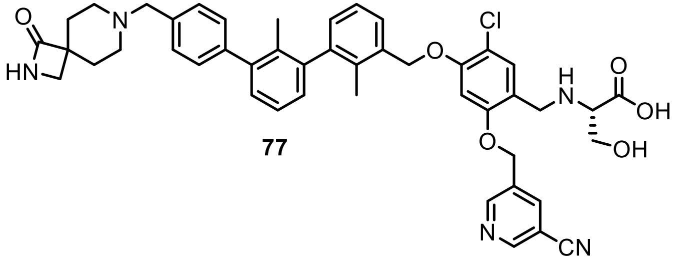 **77**	
78	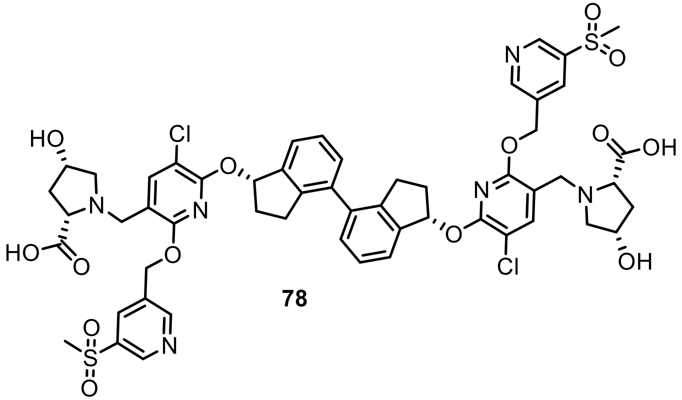 **78**	
79	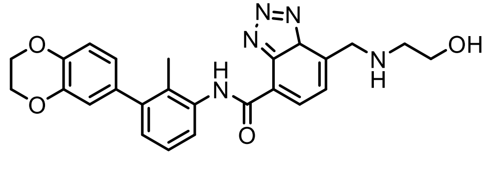 **79**	88. Qin, M.; Cao, Q.; Zheng, S.; Tian, Y.; Zhang, H.; Xie, J.; Xie, H.; Liu, Y.; Zhao, Y.; Gong, P. Discovery of [1,2,4]Triazolo[4,3-a]pyridines as Potent Inhibitors Targeting the Programmed Cell Death-1/Programmed Cell Death-Ligand 1 Interaction. *J. Med. Chem.* **2019**, *62* (9), 4703–4715.

80	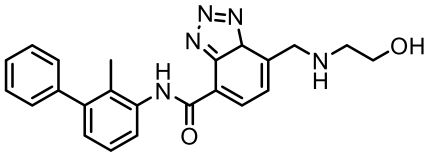 **80**	
81	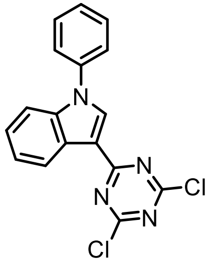 **81**	89. Patil, S.P.; Fink, M.A.; Enley, E.S.; Fisher, J.E.; Herb, M.C.; Klingos, A.; Proulx, J.T.; Fedorky, M.T. Identification of Small-Molecule Inhibitors of PD-1/PD-L1 Protein-Protein Interaction. ChemistrySelect 2018, 3, 2185–2189
82	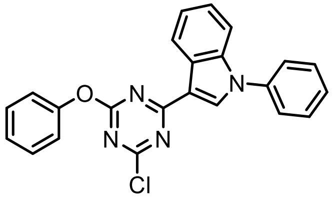 **82**	
83	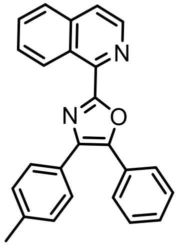 **83**	

84	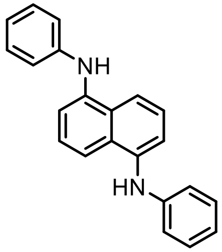 **84**	
85	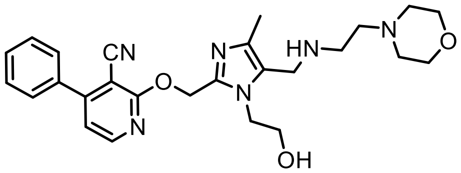 **85**	91. Dömling, A. Inhibitors of the PD-1/PD-L1 protein/protein interaction., 13 July 2017, WO2017/118762 A1<br>92. Dömling, A. 3-cyanotiphene derivatives as inhibitors of the PD-1/PD-L1 interaction.,10 January 2019, WO2019/008152 A1<br>93. Dömling, A. 3-(azolylmethoxy)biphenyl derivatives as inhibitors of the PD-1/PD-L1 protein-protein interaction, 10 January 2019, WO2019/008154 A1<br>94. Dömling, A. Inhibitors of the PD-1/PD-L1 interaction, 10 January 2019, WO2019/008156 A1
86	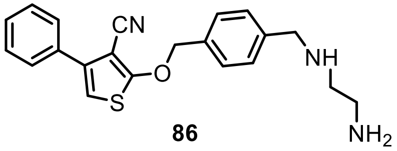 **86**	
87	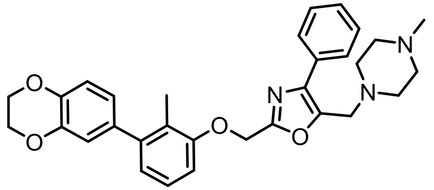 **87**	
88	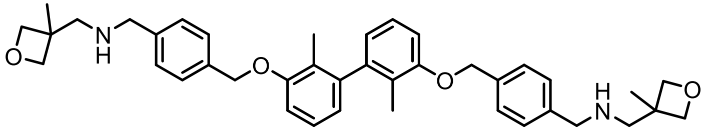 **88**	

89	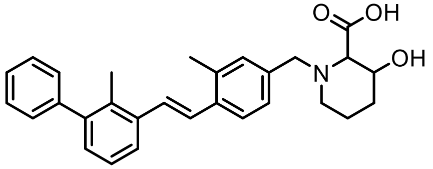 **89**	95. Wang, Y.; Xu, Z.; Wu, T.; He, M.; Zhang, N. 2018, Aromatic acetylene or aromatic ethylene compound, intermediate, preparation method, pharmaceutical composition and use thereof, 11 January 2018. WO2018006795
90	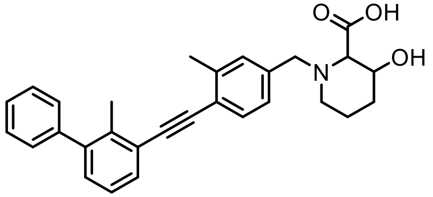 **90**	
91	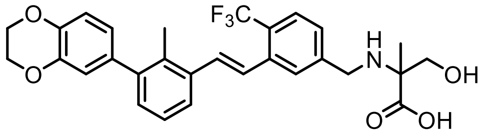 **91**	
